# Heterosexual, gay, and lesbian people’s reactivity to virtual caresses on their embodied avatars’ taboo zones

**DOI:** 10.1038/s41598-021-81168-w

**Published:** 2021-01-26

**Authors:** Martina Fusaro, Matteo P. Lisi, Gaetano Tieri, Salvatore Maria Aglioti

**Affiliations:** 1 Sapienza, Università degli Studi di Roma & CLNS@Sapienza, Istituto Italiano Di Tecnologia, Rome, Italy; 2 Social Neuroscience Laboratory, IRCCS Fondazione Santa Lucia, Rome, Italy; 3 Virtual Reality Lab, University of Rome Unitelma Sapienza, Rome, Italy

**Keywords:** Psychology, Human behaviour, Sexual behaviour, Social neuroscience

## Abstract

Embodying an artificial agent through immersive virtual reality (IVR) may lead to feeling vicariously somatosensory stimuli on one’s body which are in fact never delivered. To explore whether vicarious touch in IVR reflects the basic individual and social features of real-life interpersonal interactions we tested heterosexual men/women and gay men/lesbian women reacting subjectively and physiologically to the observation of a gender-matched virtual body being touched on intimate taboo zones (like genitalia) by male and female avatars. All participants rated as most erogenous caresses on their embodied avatar taboo zones. Crucially, heterosexual men/women and gay men/lesbian women rated as most erogenous taboo touches delivered by their opposite and same gender avatar, respectively. Skin conductance was maximal when taboo touches were delivered by female avatars. Our study shows that IVR may trigger realistic experiences and ultimately allow the direct exploration of sensitive societal and individual issues that can otherwise be explored only through imagination.

## Introduction

The sense of touch is essential not only for detecting the presence of a stimulus on the skin, or for haptically exploring surfaces and manipulating objects, but also for promoting affectively salient social interactions^1^. The socio-affective dimensions of touch comprise a variety of behaviors like physical contact between individuals as well as the development of affiliative and interpersonal links^2^ that fundamentally contribute to shaping relationships in both non-human^3^ and human primates^4^. Mother-newborn interactions and romantic relationships, for example, clearly indicate that skin-to-skin contact promotes bonding between individuals. The bodily regions where one may touch other individuals within or outside one’s social network are relation-specific, as indicated by studies in which topographic bodily maps of touch appropriateness were derived from self-reports of healthy humans who answered “who can touch you where” questions^5^. Specifically, 1368 participants from different western countries (Finland, France, Italy, Russia, and the UK) were asked to color the bodily areas on a picture of a human mannequin where they would allow different others, ranging from their partner to total strangers, to touch them. The self-report responses were combined to create color maps, showing that the taboo zones evoked by the mental imagination of touch overlap with those expected to be elicited by actual touch. Moreover, the distribution of these zones is modulated by the relationship between the toucher and the receiver.

In 2014, Turnbull and colleagues^6^ presented the first quantitative and systematic investigation of erogenous zones with the aim of testing the overlap between the distribution of erogeneity around the body and the arrangement of the body parts in the somatosensory cortex. By means of a survey, they collected data in which participants had to rate the erogenous intensity of different body parts. However, results demonstrated that the somatosensory theory was poorly supported. A recent paper by Maister and colleagues^7^ expanded on these results exploring through an online survey the intensity of sexual arousal related to different body parts, on the respondent’s body and on a imagined partner’s body, in response of being touched or being looked at. Interestingly, their results showed the existence of topographic arousal shared between the own and partner’s body as well as between touch and vision.

Social psychology studies about the role that gender plays in touching behavior show that for heterosexual participants, touches coming from same-sex persons are generally viewed with unease and anxiety^8^. This is particularly true when the touching person is a stranger, and the touch stimulus has intimate connotations due to the touched body part or the context in which it is delivered^9^.

**Figure 1:**
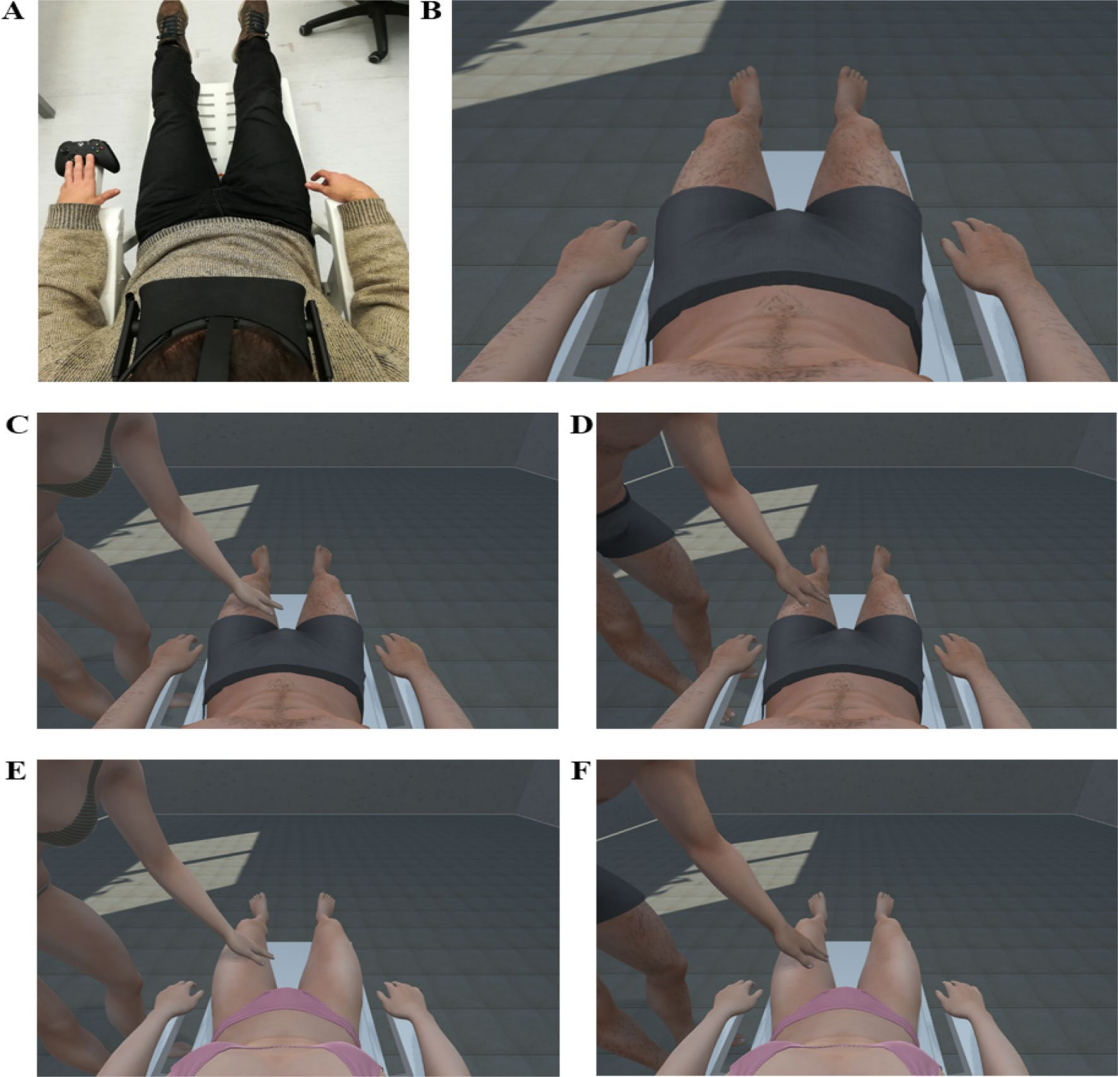
Participants were seated on a beach chair (**A**) and observed, through an HMD, a virtual body having the same position as their real one (**B**). Example of a female (**C**–**E**) or a male (**D**–**F**) avatar touching the participants’ gender-matched virtual bodies. The virtual environment was implemented on the Unity3D platform (http://unity3d.com/unity) and animation-enabled models of female and male virtual bodies were created with Iclone 7 (https://www.reallusion.com/iclone/) and Autodesk MotionBuilder (https://www.autodesk.com/products/motionbuilder/overview) and were customized appropriately for the purposes of the study using 3D Studio Max 2017 (http://www.autodesk.es/products/3ds-max/overview).

Psychology and neuroscience studies indicate that the feeling of ownership^10^ (the sense that our body belongs to us) plays a fundamental role in developing body awareness. Expanding on the induction of illusory ownership over physical body parts [i.e., like rubber hand^11^, full body^12^;or even faces,^13^], studies show that ownership over virtual body parts can be easily induced using immersive virtual reality—IVR^14^. More specifically, by using a head-mounted display (HMD) it is possible to substitute the participant’s real body with a virtual one having the same dimensions, gender and shape, and thus investigate the bodily and brain mechanisms underpinning bodily awareness^15,16^. Tellingly, the simple passive observation of a virtual body seen from a first-person perspective (1PP) is a sufficient and necessary condition to elicit an illusory feeling of ownership over the virtual body^17–21^. So far, the effects of touch in IVR have been mainly investigated either providing a tactile stimulation independent from the visual information^22^, or pairing the tactile and the visual stimulation^23^. However, recent evidence shows that the mere observation of a pleasant (e.g., a virtual caress) or unpleasant (e.g., a virtual syringe penetrating the skin) stimuli on an embodied virtual body can induce vicarious sensations^24,25^. Therefore, IVR may represent a powerful tool for investigating behavioral, bodily, and brain reactivity to pleasant or unpleasant stimuli entering the virtual peripersonal space and approaching one’s virtual body. What remains unknown is the degree to which vicarious sensations reflect different real-life circumstances in which actual touches may have positive or dramatically negative impacts on real people. To address this question, we designed a novel IVR task in which heterosexual and gay men and heterosexual and lesbian women embodied a gender-matched virtual body and passively observed a same- or opposite-sex avatar caressing different parts of their virtual body (see Fig. 1 and Video S1). The task allowed us to test two main hypotheses. The first concerns whether in IVR vicarious touch could elicit specific patterns of behavioral and physiological reactivity depending on the touched body part (e.g. the hands, coded as a social region, or the genitals and breast, coded as intimate regions). We expected that touches on intimate areas would elicit higher erogeneity and arousal, paired with an enhancement in skin conductance responses and a slow-down in the heart rate; moreover, we expected that touches on the social area would be rated as the most appropriate and pleasant. The second hypothesis is that the sexual orientation of female and male participants has an influence on the reactivity to touch. The results allowed us to obtain novel information on “who can touch my virtual body and where” in terms of appropriateness and erogeneity (as well as of arousal and [un]pleasantness, see Supplemental Information). Thanks to our paradigm we not only expanded on studies on imagination of the effect of being touched^5,7,26^ but we also gauged information potentially useful for dealing with sensitive issues that impact real life (e.g. sexual harassment) which, for obvious ethical reasons, cannot be explored by direct stimulation of real bodies.

## Results

### Study 1

Based on a pilot study performed on an independent group of 74 participants, the body parts were classified as intimate, social, or neutral (see Figure S1 and Table S1).

For each dependent variable [namely, the scores for each of the four in-session VAS questions, the skin conductance response (SCR) in µS and heart rate (HR), in a time window of 6 s post-stimulus] we performed a linear mixed-effects analysis in R^27^. As fixed effects, all models had the main effects and the interactions between all our experimental factors—Gender (men and women), Touching avatar (male and female), Area (intimate, social, neutral). For the questions at the end of the block, we built two linear mixed-effects models: one had the body ownership ratings as outcome and the type of question (embodiment, control), the Touching Avatar and Gender as predictors; the other had the vicarious touch ratings as outcome and the Touching Avatar and Gender as predictors. In all models, participants were always set as a random effect, with correlated intercept and slopes for all our fixed effects varying among this random effect. A simpler random effect structure was set when the model failed to converge. Post-hoc pairwise comparisons were conducted with the Tukey’s method. The syntax for all the models and additional details regarding the analyses can be found in the Supplemental information (in the section “Mixed Effects Models Syntax and Details”).

### Body ownership questionnaire

The two embodiment questions (items 1–2, in Supplemental Information) and the two control questions (items 3–4) were averaged for each block (male touch vs. female touch; aggregation methods are indicated in^28^. The model (R^2^_marginal_ = 0.232, R^2^_conditional_ = 0.665) revealed a main effect of the embodiment factor (χ2(1) = 38.65, *p* < 0.001), accounted for by higher values in the embodiment questions (estimate 27.9, SE 4.6, t.ratio 6.607, *p* < 0.001), than the control ones (mean 58.2, SE 3.30, lower CL 51.6, upper.CL 64.9 vs. mean 30.3, SE 3.36, lower CL 23.5, upper.CL 37.1, Figure S2 and Table S2), suggesting that participants were incorporating the virtual body. No other significant results were found.

### Vicarious touch

The analysis of the subjective reports concerning the illusory sensation of feeling touches over one’s own body (R^2^_marginal_ = 0.031, R^2^_conditional_ = 0.714) did not reveal any significant effect (Figure S2 and Table S2). There were no differences in vicarious touch sensations depending on the touching avatar neither among women (Female Touching Avatar mean: 42.6, SE 4.75, lower.CL 33.1, upper.CL 52.1; Male Touching Avatar mean: 45.6, SE 4.75, lower.CL 36.1, upper.CL 55.1), nor among men (Female Touching Avatar mean: 40.8, SE 4.75, lower.CL 31.3, upper.CL 50.3; Male Touching Avatar mean: 35.2, SE 4.75, lower.CL 25.7, upper.CL 44.7) and there were no differences between the two groups, suggesting that the participants perceived similarly touches from both avatars. In a study by Ward and colleagues^29^, participants had to rate the intensity of the sensation of observing a human touch (vs. object and dummy). Results showed that people with a low level of mirror-touch synesthesia rated the vicarious human touch around 1.5–2 while individuals with mirror-touch synesthesia rated the sensations around 4 (on a scale from 0 to 10). It is worth noting that the average intensity in our task is around 40, which is similar to what experienced by synesthetes judging intensity of vicarious touch on others.

### Correlation between ownership and vicarious touch

Pearson’s correlation analysis was used to assess any relations between the Embodiment, the Vicarious Touch, and the Control Items for Embodiment. *P*-values were corrected with the Holm’s procedure. The analysis revealed a significant positive correlation between Embodiment and Vicarious Touch (r = 0.458; Table S17).

### Appropriateness

**Figure 2:**
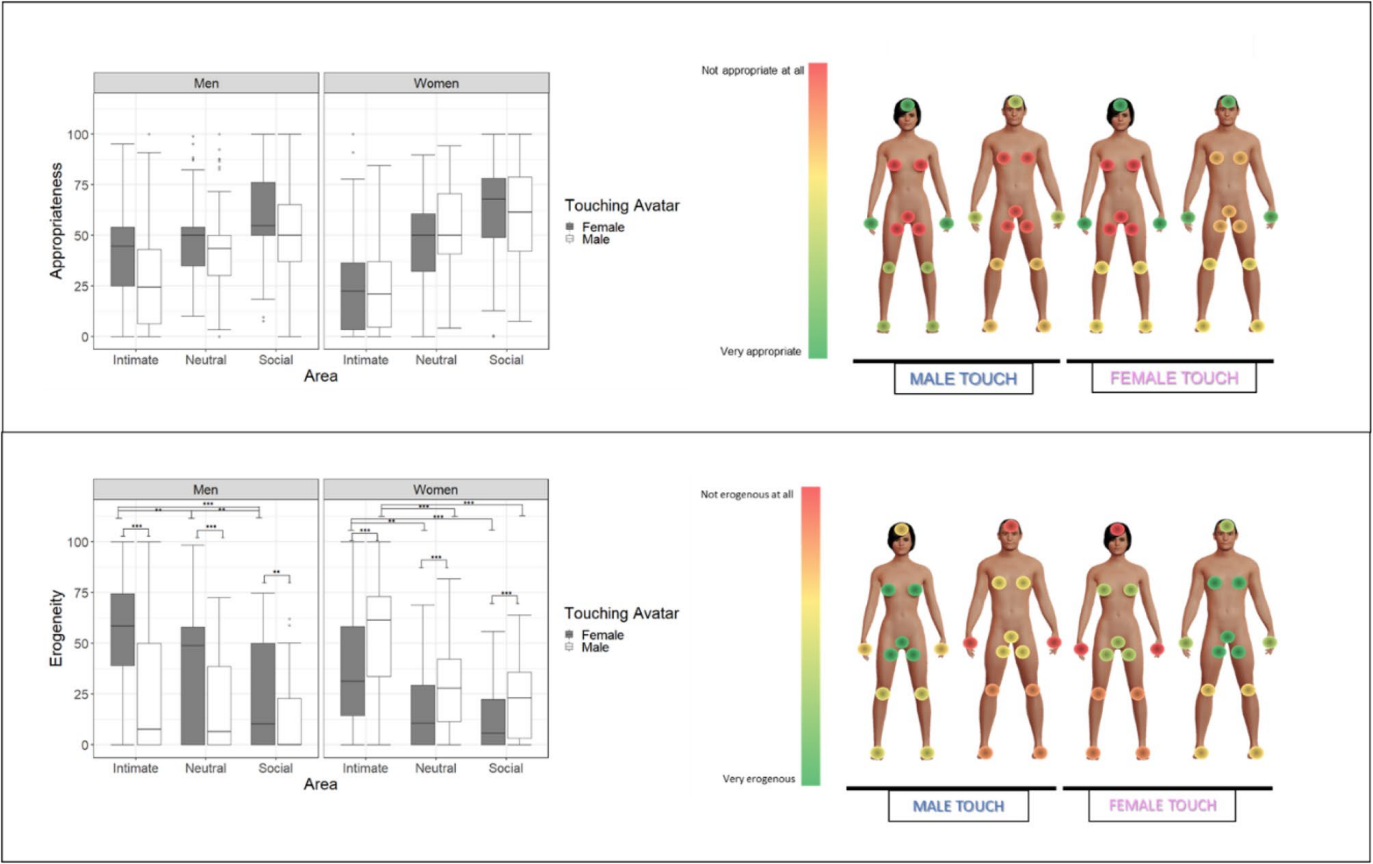
Study 1 (Heterosexual men and women). On the left of the figure the two boxplots regarding Appropriateness and Erogeneity for the interaction between Gender, Touching avatar and Area. On each box, the central mark indicates the median, and the lower and upper hinges correspond to the 25th and 75th percentiles. The upper whisker extends to the largest value no further than 1.5 * IQR (inter-quartile range) from the hinge. The lower whisker extends to the smallest value at most 1.5 * IQR of the hinge. Points outside this range are shown individually. Signif.codes: ‘***’ 0.001 ‘**’ 0.01 ‘*’ 0.05 ‘*’. On the right of the figure, mean values for Appropriateness and Erogeneity depending on the Touching avatar (male on the left and female on the right). The dark red represents the lowest mean, while the dark green represents the highest mean. The virtual bodies used in the figure were created using MakeHuman (http://www.makehumancommunity.org/).

The multilevel linear regression on participants’ subjective feelings of appropriateness (R^2^_marginal_ = 0.289, R^2^_conditional_ = 0.575) yielded a main effect of the Touching avatar and of the Area along with three 2-way interactions (the model, all the effects and the averages for each conditions are shown in Fig. 2 and in Tables S2 and S3). The interaction between Gender and Touching avatar (χ2(1) = 29.71, *p* < 0.001) revealed that touches delivered to men by the female avatar were considered more appropriate than touches delivered by the male avatar (estimate = 8.6, SE = 1.42, *p* < 0.001). Women considered as equally appropriate touches delivered by male and female avatars.

The post-hoc comparisons of the interaction Gender and Area (χ2(2) = 16.67, *p* < 0.001) showed that the main effect of the Area was not modulated by the Gender of the participant: for both men and women, the social area was considered more appropriate to be touched compared to the neutral (men: *p* = 0.008; women *p* = 0.003) and the intimate (men:*p* < 0.001; women: *p* < 0.001) ones; moreover, the neutral area was considered as more appropriate than the intimate one (men: *p* < 0.001, women: *p* < 001; see Table S3).

The significance of the interaction Touching avatar and Area (χ2(2) = 11.15, *p* = 0.003) was accounted for by higher appropriateness of the female than the male touch on the intimate area (estimate = 6.947, SE = 1.51, *p* < 0.001).

### Erogeneity

The linear mixed effects model (R^2^_marginal_ = 0.258, R^2^_conditional_ = 0.670) revealed a main effect of the Touching avatar and of the Area along with two 2-way interactions and one triple interaction (see Fig. 2 and Tables S2 and S4). The interaction between Gender, Touching avatar and Area (χ2(2) = 38.71, *p* < 0.001) showed that touches in all the areas elicited higher erogeneity when men and women received the touch from the avatar that matched their (hetero)sexual orientation (intimate: male vs. female on men *p* < 0.001, on women *p* < 0.001; neutral: male vs. female on men *p* < 0.001, on women *p* < 0.001; social: male vs female on men *p* = 0.006, on women *p* < 0.001, see Table S4). Moreover, in both men and women touches from the opposite-sex (touching) avatar on the intimate area were always considered more erogenous than touches on neutral (female on men: *p* = 0.006; male on women *p* < 0.001) and on social (female on men: *p* < 0.001; male on women: *p* < 0.001), higher erogeneity on neutral compared to social in men (*p* = 0.002), while no differences were found between social and neutral in women (*p* = 0.88). When the touches were delivered from same-sex avatar, among men there were no differences in the erogeneity between social and neutral areas (*p* = 0.94), intimate and social (*p* = 0.35), and intimate and neutral (*p* = 0.83), while among women higher erogeneity was reported in intimate area than in neutral and social (respectively, *p* = 0.006, *p* < 0.001) and no differences between social and neutral (*p* = 0.86).

The results concerning (un)pleasantness and arousal (Table S5, Figure S3; Table S6, Figure S4, respectively) also revealed that touches were considered more or less pleasant and arousing depending on the area and the gender of the toucher. Ratings of pleasantness and arousal showed a pattern similar to that which was found for appropriateness and erogeneity, respectively. Due to space limitations, these data are reported in the Supplemental Information.

### Skin conductance responses (SCR)

**Figure 3:**
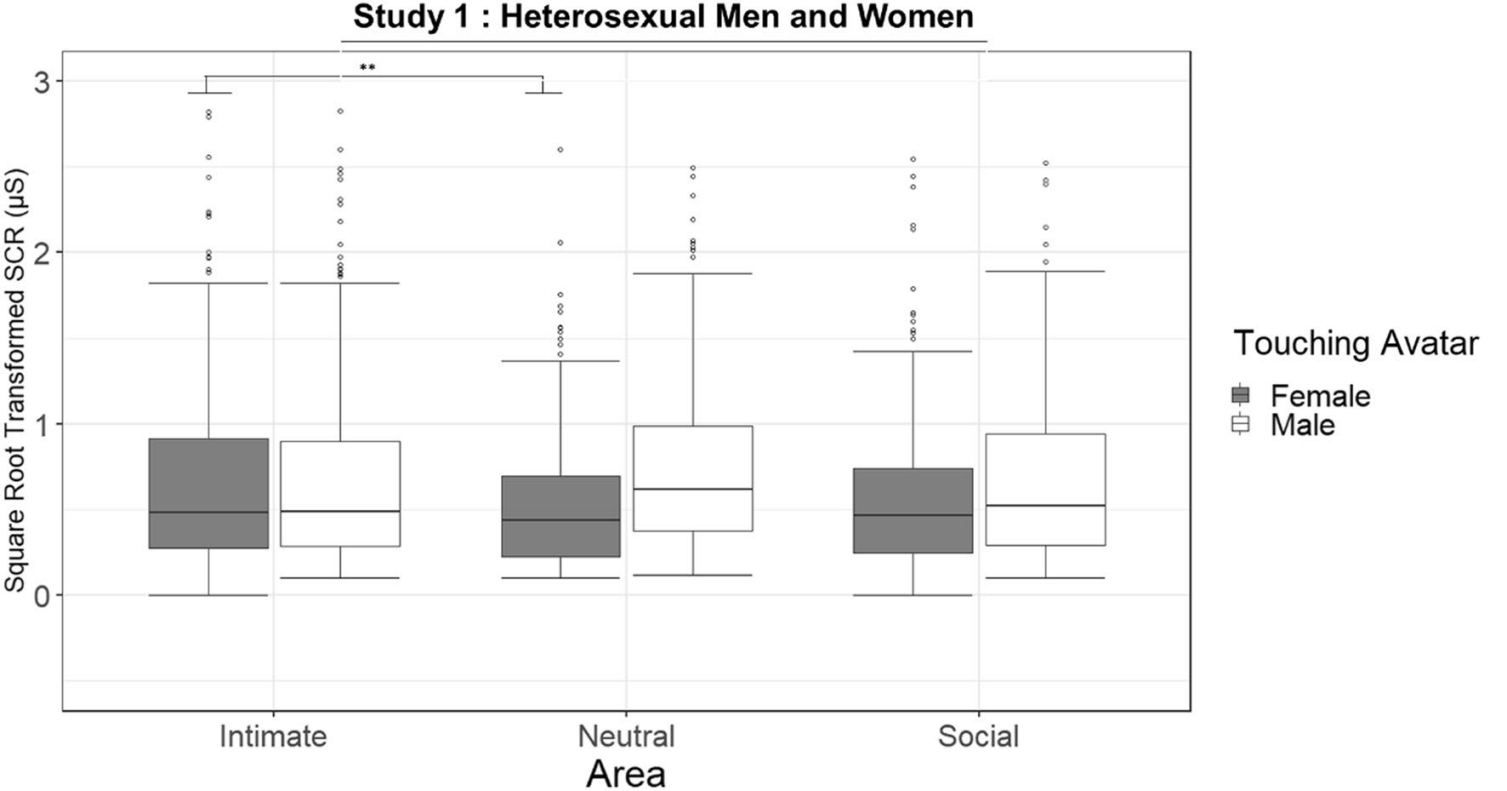
Study 1 (Heterosexual men and women participants): Boxplot of the square root-transformed SCRs for the interaction between Touching avatar and Area. On each box, the central mark indicates the median, and the lower and upper hinges correspond to the 25th and 75th percentiles. The upper whisker extends to the largest value no further than 1.5 * IQR (inter-quartile range) from the hinge. The lower whisker extends to the smallest value at most 1.5 * IQR of the hinge. Points outside this range are shown individually. Signif. codes: ‘***’ 0.001 ‘**’ 0.01 '*' 0.05 ‘*’.

The linear mixed effects model (R^2^_marginal_ = 0.043, R^2^_conditional_ = 0.556) revealed a main effect of the Touching avatar and two 2-way interactions (Table S2 and Fig. 3). The interaction between the Gender and the Area (χ2(2) = 6.60, *p* = 0.036; Table S7) revealed that SCRs were enhanced in men observing intimate compared to neutral (estimate = 0.11, SE = 0.03, *p* = 0.02) touch, while in women we did not find differences between the areas. The interaction between the Touching avatar and the Area (χ2(2) = 13.61, *p* < 0.005; Table S7) was determined by the significant increase during the female touch on intimate compared to neutral (*p* = 0.001), and no other significant comparisons were found.

### Heart rate (HR)

The model (R^2^_marginal_ = 0.058, R^2^_conditional_ = 0.821) did not show any modulation of this measure contingent upon the Touching Avatar and the Area (see in the Table S2). A trend towards significance (*p* = 0.06) was found for the gender and for the interaction between Gender and Area (*p* = 0.06). However, post-hoc comparisons did not show significant results (see Table S8).

### Study 2

42 participants were classified as lesbian women (21) and gay men (21) using the sexual orientation Kinsey scale. The participants completed the same Study 1 IVR task with the same procedure (Fig. 1).

### Body ownership questionnaire

The model (R^2^_marginal_ = 0.254, R^2^_conditional_ = 0.706) showed a main effect of the embodiment factor (χ2(1) = 32.65, *p* < 0.001) which was accounted for by higher values in the embodiment questions (estimate 27.9, SE 5, *p* < 0.001, Figure S5), than the control ones (mean Embodiment 58.8, SE 2.89, lower CL 53, upper.CL 64.6 vs. mean Control 30.9, SE 3.62, lower.CL 23.6, upper.CL 38.2). No other significant effect was found.

### Vicarious touch

The analysis (R^2^_marginal_ = 0.045, R^2^_conditional_ = 0.885) revealed a significant interaction between Touching avatar and participants’ Gender (χ2(1) = 5.655, *p* = 0.01; Figure S5, Table S9), driven by a slightly higher, but not significant, level of illusion among women during the female touch (estimate 4.71, SE 2.42, *p* = 0.22) compared to the male touch (mean Female Touching avatar 47.6, SE 4.74, lower CL 38.1, upper.CL 57.2 vs. mean Male Touching avatar 42.9, SE 4.74, lower CL 33.4, upper CL 52.4). Among men there were similar levels of illusion across the two blocks (mean Female Touching avatar 52.1, SE 4.83, lower.CL 42.5, upper.CL 61.8 vs. mean Male Touching avatar 55.3, SE 4.83, lower.CL 45.6, upper.CL 65.0). Overall, in the non-heterosexual sample the mean vicarious touch ratings (49.06 ± 21.71) were comparable to those found in the heterosexual sample (Study 1).

### Correlation between ownership and vicarious touch

The correlation analysis revealed a significant positive association between Embodiment and Vicarious Touch (r = 0.562; Table S17) and a negative association between Vicarious Touch and Control Questions for Embodiment (r = − 0.384).

### Appropriateness

**Figure 4:**
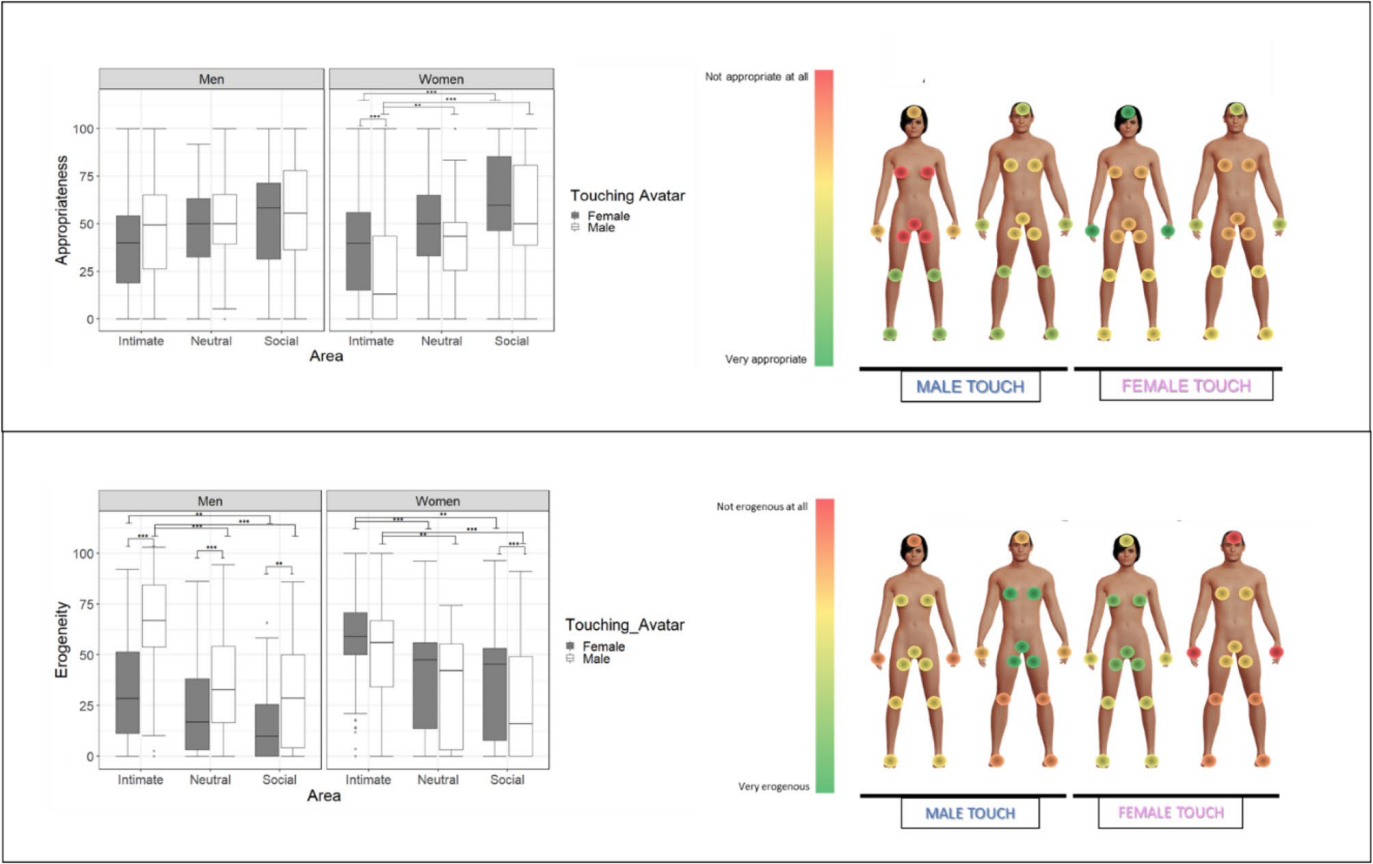
Study 2 (Gay men and Lesbian women participants): On the left of the figure the two boxplots regarding Appropriateness and Erogeneity for the interaction between Gender, Touching avatar and Area. On each box, the central mark indicates the median, and the lower and upper hinges correspond to the 25th and 75th percentiles. The upper whisker extends to the largest value no further than 1.5 * IQR (inter-quartile range) from the hinge. The lower whisker extends to the smallest value at most 1.5 * IQR of the hinge. Points outside this range are shown individually. Signif.codes: ‘***’ 0.001 ‘**’ 0.01 ‘*’ 0.05 ‘*’. On the right of the figure, mean values for Appropriateness and Erogeneity depending on the Touching avatar (male on the left and female on the right). The dark red represents the lowest mean, while the dark green represents the highest mean. The virtual bodies used in the figure were created using MakeHuman (http://www.makehumancommunity.org/).

The multilevel linear regression (R^2^_marginal_ = 0.119, R^2^_conditional_ = 0.537) yielded a main effect of the Touching avatar and of the Area along with two 2-way interactions and the 3-way interaction (Fig. 4,Table S9). The triple interaction between Gender, the Touching avatar and the Area (χ2(2) = 8.7227, *p* = 0.001, Table S10) revealed that gay men considered equally appropriate touches from the male or the female touching avatar in all the areas and that no area was considered more appropriate than the other, regardless the touching avatar’s gender. Differently, lesbian women considered more appropriate touches on intimate area from a female avatar compared to the male avatar (*p* < 0.001) but equally appropriate on social and neutral (*p* = 0.56, *p* = 55, respectively) from both avatars; moreover, for lesbian women, touches on neutral and social area were considered more appropriate than on intimate area (*p* = 0.01 and *p* < 0.001) when touched by a male, and no differences between the former two (*p* = 0.08). When lesbian women were touched by the female avatar, the social area was considered more appropriate than the intimate one (*p* < 0.001) and no other differences were detected between other areas.

### Erogeneity

The linear mixed effects analysis (R^2^_marginal_ = 0.241, R^2^_conditional_ = 0.592) revealed a main effect of the Touching avatar and of the Area along with two 2-way interactions and the triple interaction (Table S9). The interaction between Gender, Touching avatar and Area (χ2(2) = 18.31, *p* < 0.001; see Fig. 4,Table S11) showed that men considered as more erogenous touch from the male than the female one, in all the areas. Differently, lesbian women considered as equally erogenous the touch from a female and a male avatar on intimate and neutral areas (*p* = 0.20 and *p* = 0.78) but more erogenous on the social when touched by the female (*p* < 0.001). The female touch on gay men was considered more erogenous in the intimate compared to the social area (*p* = 0.01); the male touch on gay men, differently, was considered more erogenous when delivered on intimate compared to neutral (*p* < 001) and social (*p* < 0.001) areas that in turn did not differ from one another (*p* = 0.98). The female touch on lesbian women was considered more erogenous on intimate than in neutral and social areas (*p* = 0.001 and *p* = 0.002) and equally erogenous on neutral and social; the male touch on lesbian women elicited the same pattern of results as the female touch (intimate more erogenous than neutral, *p* = 0.003 and than social, *p* < 0.001; no differences between neutral and social = 0.42).

The results concerning (un)pleasantness and arousal (Table S12, Figure S6; Table S13, Figure S7, respectively) are reported in the Supplemental Information.

### SCR

**Figure 5:**
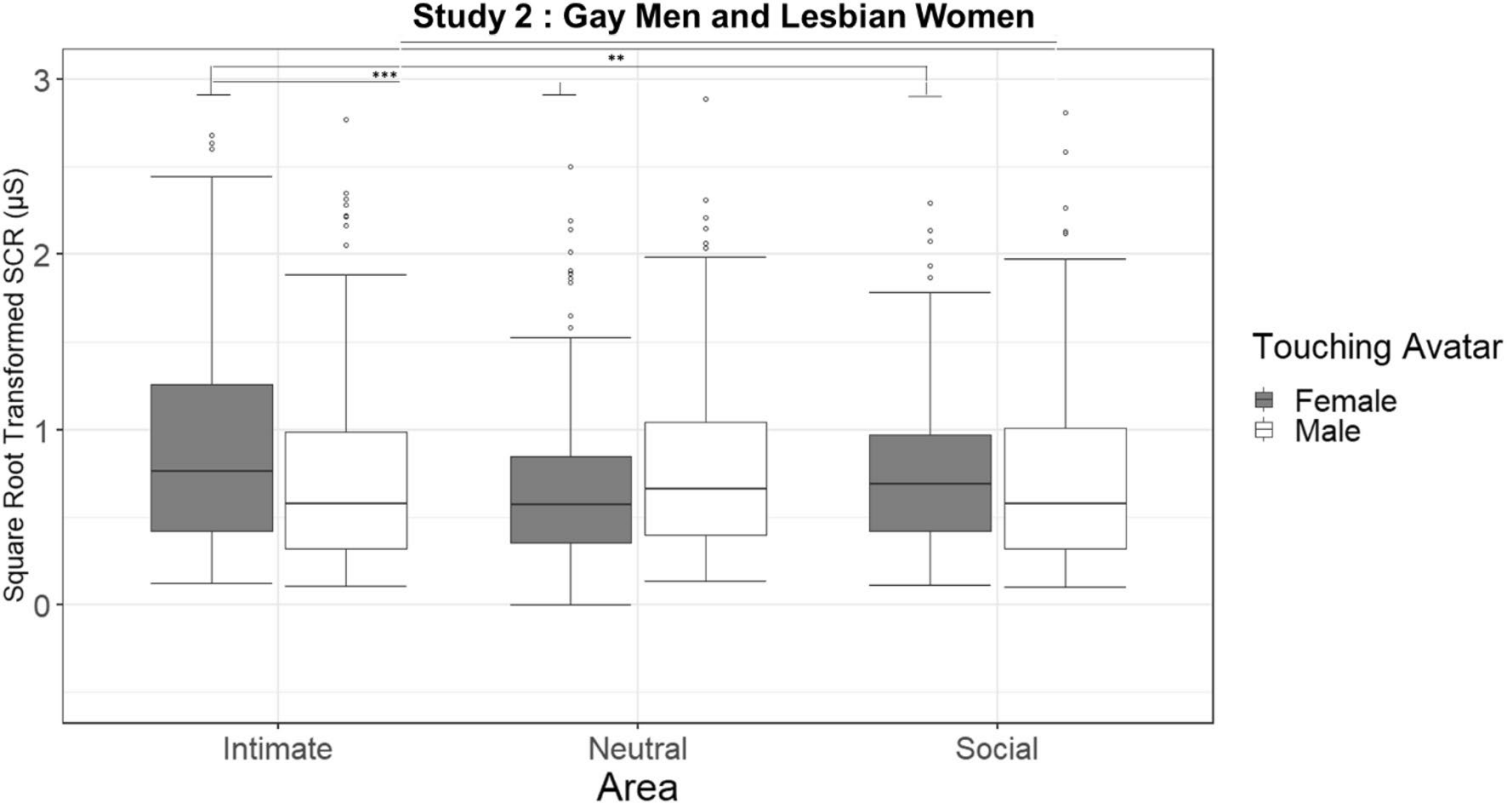
Study 2 (Gay men and Lesbian women): Boxplot of the square root-transformed SCRs for the interaction between Touching avatar and Area. On each box, the central mark indicates the median, and the lower and upper hinges correspond to the 25th and 75th percentiles. The upper whisker extends to the largest value no further than 1.5 * IQR (inter-quartile range) from the hinge. The lower whisker extends to the smallest value at most 1.5 * IQR of the hinge. Points outside this range are shown individually. Signif. codes: ‘***’ 0.001 ‘**’ 0.01 ‘*’ 0.05 ‘*’.

The linear mixed effects analysis (R^2^_marginal_ = 0.018, R^2^_conditional_ = 0.540) revealed a main effect of the area and a 2-way interaction between the Touching avatar and the Area (Table S9 and Fig. 5). The interaction (χ2(2) = 19.28, *p* < 0.001; see Table S14) revealed significant increase during the female touch on intimate compared to neutral area (*p* < 0.001) and to social (*p* = 0.003), while no other differences were found.

### HR

The model (R^2^_marginal_ = 0.012, R^2^_conditional_ = 0.827) revealed a significant interaction between the Touching avatar and the Area (χ2(2) = 10.88, *p* = 0.004) driven by a slightly higher, but not significant, number of heart beats following the female touch delivered to the intimate (estimate 0.12, SE 0.05, t.ratio 2.169, *p* = 0.25) compared to the neutral area(mean Intimate 7.99, SE 0.18, lower.CL 7.61, upper.CL 8.36 vs. mean Neutral 7.86, SE 0.18, lower.CL 7.48, upper.CL 8.24). No other significant difference was found (see Table S9 and S15).

## Discussion

Conducting a scientific study in which real people are actually touched on a variety of body regions, including taboo areas, by someone who is not the romantic partner, is clearly not possible for fundamental ethical, psychological, and societal reasons. We devised a novel IVR-based paradigm in order to explore whether human-avatar interactions in virtual reality trigger mental and corporeal states that are similar to those evoked by actual human–human interactions in real life. Specifically, we explored the vicarious sensations of touches delivered to different parts of a virtual body, seen from a 1PP, and how these sensations were modulated by different factors that fundamentally shape daily life touch-mediated interactions. The results show that virtual touches can induce sensations that may be reminiscent of what could happen when one is caressed by a stranger in real life. Notably, explicit and implicit responses to virtual touches were influenced by the touched area on the virtual body, the gender and the sexual orientation of the participants, as well as the gender of the touching avatar.

The conscious experience of tactile sensations, induced by seeing someone else being tactually stimulated, characterizes mirror-touch synaesthesia^29,30^. While in its full form this condition is rare^31^, vicarious touch has been reported in the neurotypical population as a consequence of exposure to pictures^32^ or videos^33,34^ depicting tactile stimuli delivered to others. Relevant to the present study is that IVR seems to be particularly adept at inducing feelings of vicarious touch, contingent upon the observation of stimuli delivered to an avatar seen from 1PP, a condition that highly favours embodiment^20^. The majority of IVR studies have explored the effect of threatening events on vicarious somatosensory feelings^35,36^ and only recently the effect of pleasant stimuli on a virtual body has been investigated^23^. Tellingly, the within-subject exploration of seeing both painful and pleasant virtual stimuli delivered to an embodied avatar revealed that vicarious pleasant touch may also be induced^24,25^. Moreover, in keeping with studies on pleasant touch delivered to a rubber hand^37^, we have been able to induce a strong illusion of ownership over the virtual hand, contingent upon pleasant stimuli^25^. In contrast to previous important studies investigating the appropriateness of social touch through imagination or questionnaires^5–7,26^, our paradigm allowed us to directly test the sense of body ownership, which is an important component of touch perception^38^. Indeed, the positive correlation between the feeling of ownership over the virtual body and the reported intensity regarding the vicarious touch hints at the interplay between these two phenomena. Moreover, studies based on questionnaires do not investigate the contribution of embodiment to different sensations arising from the avatar’s body as if it were one’s own actual body. Thus, the derivative notion of self-related sensations from what is seen on the avatar represents a step ahead in the creation of new setups for obtaining highly controlled, yet ecological, situations that are virtually similar to real-life, in cases in which the issues cannot be investigated without violating individual space and social rules. Future studies are needed to systematically explore these sensations and to better understand the mechanisms that trigger this type of vicarious perception.

Even though the skin is a complex organ including many types of afferents, studies indicate that activation of the C Tactile system—that is considered of fundamental importance for social touch-correlates with subjective ratings of touch pleasantness^39^. Crucially, however, perceptual valence and pleasantness do not depend solely on afferent sensations^40^. Indeed, top-down variables that influence salience, appropriateness, and pleasantness (e.g., the relationship between toucher and touched, the context, social status) play a key role^41^. Among the top-down factors that modulate reactions to touch, the gender of the toucher may be one of the most important. Studies suggest that female touch occurs more frequently under daily life circumstances^42^ and is more accepted by both men and women^5^. Moreover, women engage in and are comfortable with more intimate forms of same-sex touch than men^43^. In a study by Scheele and colleagues^44^ authors tested the reward of touch (independently from the intensity and the actual cutaneous stimulation) in a sample of heterosexual men which believed they were touched by a men or a women (while the touch was always coming from a woman). Their results showed that oxytocin could increase the pleasantness of the female touch and not the male one and that this effect negatively correlated with the autistic-like traits. Overall, this gender difference could be explained by looking at the animal kingdom, in which grooming occurs predominantly between females^45^. Tellingly, heterosexual men tend to inhibit same-sex contact, which is considered by them as sexual involvement^8,46^, probably to comply with their fear of being perceived as gay^9^. The male- and female-looking touching avatars used in the present study allowed us to directly test the effect of toucher’s gender on reactions to pleasant and intimate touch. Moreover, we have been able to explore, directly, the issue of how the participants’ sexual orientation drives ratings of the appropriateness of touch coming from the same or different gender. Overall, a clear role for sexual orientation emerged. Indeed, heterosexual men considered female touch more appropriate than the male touch while gay men found same- and opposite-sex touches equally appropriate. Heterosexual women considered equally appropriate same- and opposite-gender touch while lesbian women considered same-gender touches in the intimate area as more appropriate. Interestingly, appropriateness was highest for the social and lowest for the intimate regions and, overall, (un)pleasantness significantly correlated with appropriateness (see the Supplemental Information, Table S16). These findings expand on and are consistent with what found in studies of “who can touch me where” based on imagination^5^ in western and eastern social and cultural contexts^26^.

Interpersonal touch has a strong erogenous potential, which is thought to be mediated by both CT fiber activation and the stimulated body area^47^. CT-optimal touch is not only associated with feelings of pleasantness, but also with feelings of sexual arousal. Indeed, optimal velocities (1–10 cm/s) lead to higher levels of sexual arousal than slower or faster velocities^48,49^. Similarly, Kirsch and colleagues^50^ recently tested whether a CT optimal touch (vs. a sub-optimal) could communicate specific emotions and mental states. Interestingly, the results of the study showed that CT-optimal touch was able to convey arousal, lust and desire while affiliative emotions (such as love and social support) were induced by general pleasant touch, regardless the velocity of stroking. As to the stimulated body area, the genitals generally have the highest erotogenic potential^6,47,49,51^. However, whether CT afferents, expected to be present in the hairy skin only^52^, are present in the genitals is unknown^53^. In any case, top-down factors, like the context and the relationship with the toucher, profoundly modulate sexual arousal and interact with bottom-up factors. Indeed, while a study based on imagination-driven self-reported measures indicates that sexual arousal may be triggered by stimuli on any part of the body (but only when touched during sex with the partner), the core erogenous hotspots remain the genitals, breasts, and anus^51^. Our more direct measures of different body parts’ reactivity clearly indicate that virtual touch was rated as more erogenous when delivered on intimate areas (genitals, inner thigh, breast) compared to the social and neutral areas. Reports of maximal sexual arousal were elicited when touches to the intimate areas of the supposedly embodied avatar were delivered by a virtual toucher the gender of which matched the participant’s sexual preference (see Figs. 2 and 4). In a similar vein, touches on the avatar’s intimate areas induced the highest psychophysiological reactivity of participants (see Figs. 3 and 5). Interestingly, while the effect of male touch to the intimate, social and neutral areas was similarly arousing, the touch of the female avatar was more arousing when delivered on intimate areas compared to the neutral areas. Overall, male touch elicited higher SCRs than female touch in the heterosexual participants but not in non-heterosexual participants. No effect of the gender of the participant was found, suggesting that, in both samples, arousal responses were similar across men and women. SCR is a reliable index of sympathetic activity^54^ that varies directly with reports of arousal, independently of whether the valence of the stimulus is positive or negative. It is also worth noting that inappropriate or unwanted touch can provoke anxiety^55^ and that the electrodermal system is sensitive to stimuli that elicit anxiety, particularly when no active avoidance response can be made^54^. It is thus possible that our results are affected both by sexual and non-sexual arousal. Overall, the SCRs provide objective evidence that participants were emotionally engaged, and they were responding ‘as if’ they were receiving stimuli on the virtual body but, as stated before, we cannot tease apart the role of cognitive, emotional or attentional components of the mechanism at play. No effects of virtual touches on HR were found in the two samples (see Table S2 and S9). Previous studies reported a decrease in HR following both real^56^ and virtual^25^ pleasant stroking, reflecting its positive effect and an increased parasympathetic activation. However, HR deceleration is also induced by unpleasant visual stimuli^57^. A possible explanation for not finding a consistent pattern of heart activation contingent upon stimuli to different areas may have to do with the opposite influence of the pleasant and unpleasant characteristics of the stimuli.

To the best of our knowledge, this is the first study that systematically investigates the possibility of inducing in human participants derivative sensations of intimacy, contingent upon touches delivered to an embodied avatar. Overall, these findings show that it is possible to combine the full body ownership illusion and the vicarious perception of touch to investigate intimate and social touch in a controlled experimental setting. This represents an unprecedented opportunity to explore bottom-up and top-down factors modulating the behavioral, physiological, and neural reactivity to social and intimate touch. Like for many new technologies, caveats concerning their impact at large are mandatory. While IVR may make possible exposure to situations that would be unethical or unsafe in reality, without endangering or compromising the participant’s physical integrity^58^, special attention must be paid to avoiding highly realistic IVR that may jeopardize the psychological equilibrium of participants. It has been demonstrated, for instance, that IVR could be a powerful tool to influence the perception and the beliefs of own’s gender when embodying an opposite-sex body^59^. Finally, our paradigm can be adapted flexibly to directly explore the neural underpinnings of vicarious social and intimate touch. Moreover, it may have translational implications for people with dysfunctional touching behavior (e.g., people with autism), as well as for people with neurological (e.g., people with spinal cord injuries) or psychogenic sexual dysfunctions (e.g. premature and delayed ejaculation).

## Methods

### Participants

A power analysis using More Power 6.0^60^ was conducted based on the large effect size obtained in a pilot study (30 participants, 15 women) for the interaction between Gender, Touching Avatar and Area in the analysis of the UnPleasantness ratings. Using a large effect size (ƞ2p = 0.14) and a power of 0.90 we established that 42 participants was an appropriate number. Before taking part in the study, participants rated their sexual orientation on a Kinsey scale^61^ ranging from “exclusively heterosexual” to “exclusively homosexual”, with “bisexual” in the middle of VAS (more details about how subjective ratings were collected are provided below). 44 heterosexual participants (mean ± SD in the Kinsey scale F = 90.70 ± 12.87, M = 90.64 ± 11.97) with normal or corrected visual acuity and naïve as to the purposes of the study were recruited for Study 1, with 22 women (mean age ± SD: 21.57 ± 1.54) and 22 men (23.48 ± 3.68). Two participants were excluded from the analysis (one woman and one man) due to technical issues. Moreover, 42 non-heterosexual participants (mean in the Kinsey scale F = 38.50 ± 18.67, M = 9.30 ± 10.22) took part in Study 2. As with Study 1, participants with normal or corrected visual acuity and naïve as to the purposes of the study were recruited, with 21 women (mean age: 24.05 ± 3.23) and 21 men (mean age: 26.52 ± 3.75).

### Experimental stimuli and setup

The virtual scenario was designed using 3DS Max 2017 and implemented in Unity v5.3. The virtual avatars were created using Iclone 7 and implemented in Unity. The scenario was presented by means of HMD Oculus Rift (https://www.oculus.com/). In order to realize naturalistic movements, we used Xsense motion capture suits (https://www.xsens.com/) to record the kinematics of an actor gently caressing different body parts of another actor seated on a beach chair with the right hand. The actor was instructed to perform the caress at a speed of about 3 cm/s. The actors’ kinematics were transferred on the virtual avatar by means of Motion Builder 2015 and rendered in Unity. In this way, participants observed the same kinematics implemented on the virtual touchers. Moreover, through recorded kinematics, we were able to keep the toucher’s movements constant and control for other emotional interference that can be conveyed in traditional experimental settings by confederates through other nonverbal cues^62^.

### General procedure

All participants were seated on a beach chair and wore an HMD through which they observed the virtual body from a 1PP (Fig. 1A). All the men and women that took part in the two studies observed the same designed virtual body, which was matched for the gender but not for the individual shape. The experiment was composed of two separate blocks presented in a counterbalanced order across the subjects for both groups. In one block, participants observed the touches delivered on their gender-congruent virtual body by a male avatar (Fig. 1B) and in the other block by a female avatar (Fig. 1C, see the video in the Supplemental Information). Each block consisted of 20 touches (in 10 trials the avatar was on the left and in 10 on the right). The body parts touched by the avatar were: foot, knee, thigh, genitals, belly, breast, shoulder, head, forearm, and hand. Each trial started with the observation of the participant’s own virtual body. Then the touching avatar appeared and after about 1 s caressed a given body part. The touch lasted for ~ 3 s. Participants continued to observe their own virtual body for 7000 ± 500 ms after the stimulus and, at the end of the trial (and the end of the observation), the first (of four) VAS appeared on a screen in the virtual environment, in a fixed order. Participants were asked to provide ratings about sensations elicited by the observation of the stimuli. During the experiment, galvanic skin response and ECG signals were continuously recorded. All participants were requested to look at their virtual body throughout the experiment and focus their attention on the toucher’s hand during touch.

### Subjective ratings

After each stimulus presentation, a black panel appeared in the virtual scene in front of the virtual body. The panel displayed a horizontal green line (60 cm length) with the extremities signed by “−−” and “++”. Participants were instructed to use the left hand to control a joystick that moved a vertical bar along the VAS line in order to provide their responses. Four different questions were asked: (1) “how appropriate was the touch?”, (2) “How pleasant or unpleasant was the touch?”, (3) “How arousing was the touch?”, and (4) “how erogenous was the touch?”. At the end of each of the two blocks, participants were asked to answer, using an identical VAS, four questions about the embodiment over the virtual body and one question about the sensation elicited by the vicarious touches. To check that the task instructions were understood, before wearing the HMD, participants read a description of each VAS (see supplemental information). Explanations about the constructs we wanted to investigate (erogeneity, appropriateness, pleasantness, and arousal) were provided.

### Limitations of the study

One potential limitation of the study may be that the survey to classify the different body areas was administered to a sample of respondents different from the one enrolled in the virtual reality studies. It may be noted that there may be some interindividual variability of evoked sensations and an area that is considered social for most people may be intimate for someone. Personal differences should be taken in account in future studies. Also, receiving touches on body parts might have altered the embodiment of the specific touched part. We did not record data regarding the embodiment of the single parts, which is something that is worth investigating. Moreover, future studies need to systematically investigate the neurophysiological reactivity to the observed touch in IVR. Related to this, it is worth noting that SCR and HR, being both general indices of arousal, might not have been the best measures to allow a conclusive interpretation of our results. It may be puzzling that the heart rate signals did not detect differences between touchers and areas touched. It is possible that longer recording windows, along with a baseline comparison, may be needed to better understand the psychophysiology related to the reactivity to virtual touches.

### Ethics

The experimental protocol was approved by the ethics committee of the Fondazione Santa Lucia (CE/PROG.643) and was carried out in accordance with the ethical standards of the 2013 Declaration of Helsinki. All participants gave their written informed consent to take part in the study.

## Supplementary Information

Supplementary Information.

Supplementary Video 1.

## Data Availability

All the data are available at this link: https://data.mendeley.com/datasets/8kxs53fhj4/1
