# Supplementary material for: Heterosexual, gay, and lesbian people’s reactivity to virtual caresses on their embodied avatars’ taboo zones
Source: Sci Rep. 2021 Jan 26;11:2221. doi: 10.1038/s41598-021-81168-w (PMC7838160; doi:10.1038/s41598-021-81168-w)
Supplement: Supplementary file 1 — Supplementary Information. [file 41598_2021_81168_MOESM1_ESM.docx]

**Supplemental information**

Heterosexual, gay, and lesbian people’s reactivity to virtual caresses on their embodied avatars’ taboo zones

**Martina Fusaro, Matteo Lisi, Gaetano Tieri, and Salvatore Maria Aglioti**

**Survey**

74 respondents (equal number of men and women, age 18-40) were asked to classify each of the body parts listed in the leftmost column of table S1 as belonging to one out of three possible categories scilicet social area (body parts that are used in communicate with others), intimate area ( the most intimate body parts), neutral area (body areas that cannot be classified as social or intimate). One participant was excluded from the analysis since he did not classify all the items. The Chi-Square Test of independence (χ2=710.43, df = 18, p < .0001) ran with the “crosstable” function from the R-package *gmodels* ^1^indicated the presence of a relationship between body parts and the categories. All expected frequencies were >5. Only the combination between body parts and the categories with a standardized residual greater than the critical value (4.00) were accepted as significant (at p<.0001;^2^). Inner Thigh (z=6.32), Genitals (z=8.03) and Breast (z=7.27) were significantly associated with the Intimate category; Foot (z=5.43) and Knee (z=8.86) were significantly associated with the Neutral category; Forehead (z=7.90) and Hand(z=8.93) were significantly associated with the Social category. Body parts not significantly associated with any of the three categories were excluded from the final analyses (see Table S1 and Figure S1).

|  | INTIMATE | | | NEUTRAL | | | SOCIAL | | |  |
| --- | --- | --- | --- | --- | --- | --- | --- | --- | --- | --- |
| BODY PART | % | *χ^2^* | St.  Resid. | % | *χ^2^* | St.  Resid. | % | *χ^2^* | St.  Resid. | Total Respondents |
| Foot | 23.28 | 4.13 | -2.03 | 64.38 | 29.49 | **5.43** | 12.32 | 9.03 | -3.00 | 73 |
| Knee | 6.84 | 18.60 | -4.31 | 86.30 | 78.60 | **8.86** | 6.84 | 14.65 | -3.82 | 73 |
| Inner Thigh | 83.56 | 40.03 | **6.32** | 15.06 | 5.27 | -2.29 | 1.36 | 21.64 | -4.65 | 73 |
| Genitals | 95.89 | 64.59 | **8.03** | 1.36 | 19.74 | -4.44 | 2.73 | 19.76 | -4.44 | 73 |
| Belly | 57.53 | 7.38 | 2.71 | 31.50 | 0.07 | 0.27 | 10.95 | 10.31 | -3.21 | 73 |
| Breast | 90.41 | 52.95 | **7.27** | 6.84 | 12.85 | -3.58 | 2.73 | 19.76 | -4.44 | 73 |
| Forehead | 8.21 | 17.00 | -4.12 | 6.84 | 12.85 | -3.58 | 84.93 | 62.48 | **7.90** | 73 |
| Shoulder | 6.84 | 18.60 | -4.31 | 41.09 | 3.17 | 1.78 | 52.05 | 8.78 | 2.96 | 73 |
| Forearm | 2.73 | 23.84 | -4.88 | 39.72 | 2.45 | 1.56 | 57.53 | 14.34 | 3.78 | 73 |
| Hand | 4.10 | 22.02 | -4.69 | 4.10 | 16.11 | -4.01 | 91.78 | 79.81 | **8.93** | 73 |

**Table S1.** The table shows, for each combination, the percentage of participants (n=73) that categorized the body part as intimate, social or neutral area, the chi-square contribution and the standardized residual. The significant associations between body parts and categories are marked in bold.

**
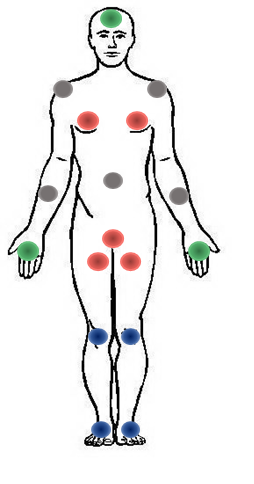
**

**Figure S1.** The figure shows all the stimulated body parts. The color indicates the area associated with the body part, as showed by the Chi-Square Test of Independence: Red (Intimate), Green (Social), Blue (Neutral), Grey (not significantly associated with any of the three possible categories). The body parts colored in Grey were excluded from the analyses.

**Embodiment questionnaire**

1)BODY OWNERSHIP: It was like I was watching my body

2)BODY OWNERSHIP: It was like the virtual body was my body

3)CONTROL (BODY OWNERSHIP): It was like I had more than one body

4)CONTROL (BODY OWNERSHIP): It was like I had not my body anymore

5) VICARIOUS TOUCH: It was like I really felt the touches on my body

**VAS**

● How much was the touch appropriate or inappropriate?

Rate how much you think the touch was appropriate on a scale ranging from 0 (totally inappropriate) to 100 (totally appropriate).

● How much was the touch pleasant or unpleasant?

Rate how much you think the touch was pleasant or unpleasant on a scale where 0, 50, and 100 represent maximal unpleasantness, neutral, and maximal pleasantness, respectively. Thus, values ranging from 0 to 49 represent an unpleasant sensation and values ranging from 51 to 100 represent a pleasant sensation.

● How much was the touch arousing?

Rate how much the touch was arousing on a scale ranging from 0 (minimal arousal) to 100 (maximal arousal).

● How much was the touch erogenous?

Rate how much the touch was sexually arousing on a scale ranging from 0 (absence of any erotic feeling) to 100 (maximal presence of erotic feelings). All the possible values can be used.

**Skin conductance responses and heart rate**

SCR and ECG were recorded throughout the experiment ^3^. An AD-Instruments PowerLab 8/35 device was used as a signal amplifier along with the ML116 GSR Amplifier (providing a 75 Hz AC excitation with low constant-voltage of 22 mVrms) with specific GSR sensors consisting of two bipolar finger electrodes. The sensors were applied to the distal phalanx of the index and middle fingers of the right hand, and the signal was sampled at 1 kHz. For the ECG, two electrodes (DORMO pre-gelled electrodes, 50 mm) were placed on the back of each hand, and the reference was placed on the left ankle. Signals were sampled at 1 kHz and filtered using a 30 Hz low-pass filter. Data were recorded and analyzed using the LabChart 7 software (AD-Instruments, Inc). The subtraction between the maximum and the minimum values of the SCR (with a minimum response of 0.01 μS) and the number of heartbeats in a time window of 6 seconds was computed ^3^. The first trigger of the time window corresponds to the start of the movement of the avatar touching the participant. To correct for non-normally distributed SCRs, the square-root transformation was computed^4^

**Study 1: Heterosexual men and women**

**Mixed Effects Model Syntax and Details**

For all models specified below (and those specified in the Study 2 section) the assumptions of linearity, homoscedasticity and normality of residuals were assessed by visual inspection. Linear mixed-effect analyses were conducted with the “lmer” function from the R-package *lme4* ^5^. P-values were computed through Type II Wald chi-square tests performed with the “Anova” function of the R-package *car* ^6^. Post-hoc comparisons were computed with the *emmeans* package ^7^.Marginal and conditional *R^2^* goodness-of-fit measures of each model ^8^ were calculated with “r.squaredGLMM” function from the R *MuMIn* package ^9^

**Appropriateness:**

*lmer(Appropriateness ~ Gender* TouchingAvatar * Area+ (1 +Area| Participant))*

**Erogeneity:**

*lmer(Erogeneity ~ Gender* TouchingAvatar * Area+ (1 +Area| Participant))*

**SCR:**

*lmer(SCR ~ Gender* TouchingAvatar * Area+ (1 +TouchingAvatar| Participant))*

**Ownership:**

*lmer(Rating ~ Gender* TouchingAvatar * Question+ (1 +Question| Participant))*

**Vicarious Touch:**

*lmer(VicariousTouch ~ Gender* TouchingAvatar+ (1| Participant))*

**UnPleasantness:**

*lmer(UnPleasantness: ~ Gender* TouchingAvatar * Area+ (1 +Area| Participant))*

**Arousal:**

*lmer(Arousal: ~ Gender* TouchingAvatar * Area+ (1 +Area| Participant))*

**HR:**

*lmer(HR ~ Gender* TouchingAvatar * Area+ (1 +TouchingAvatar| Participant))*


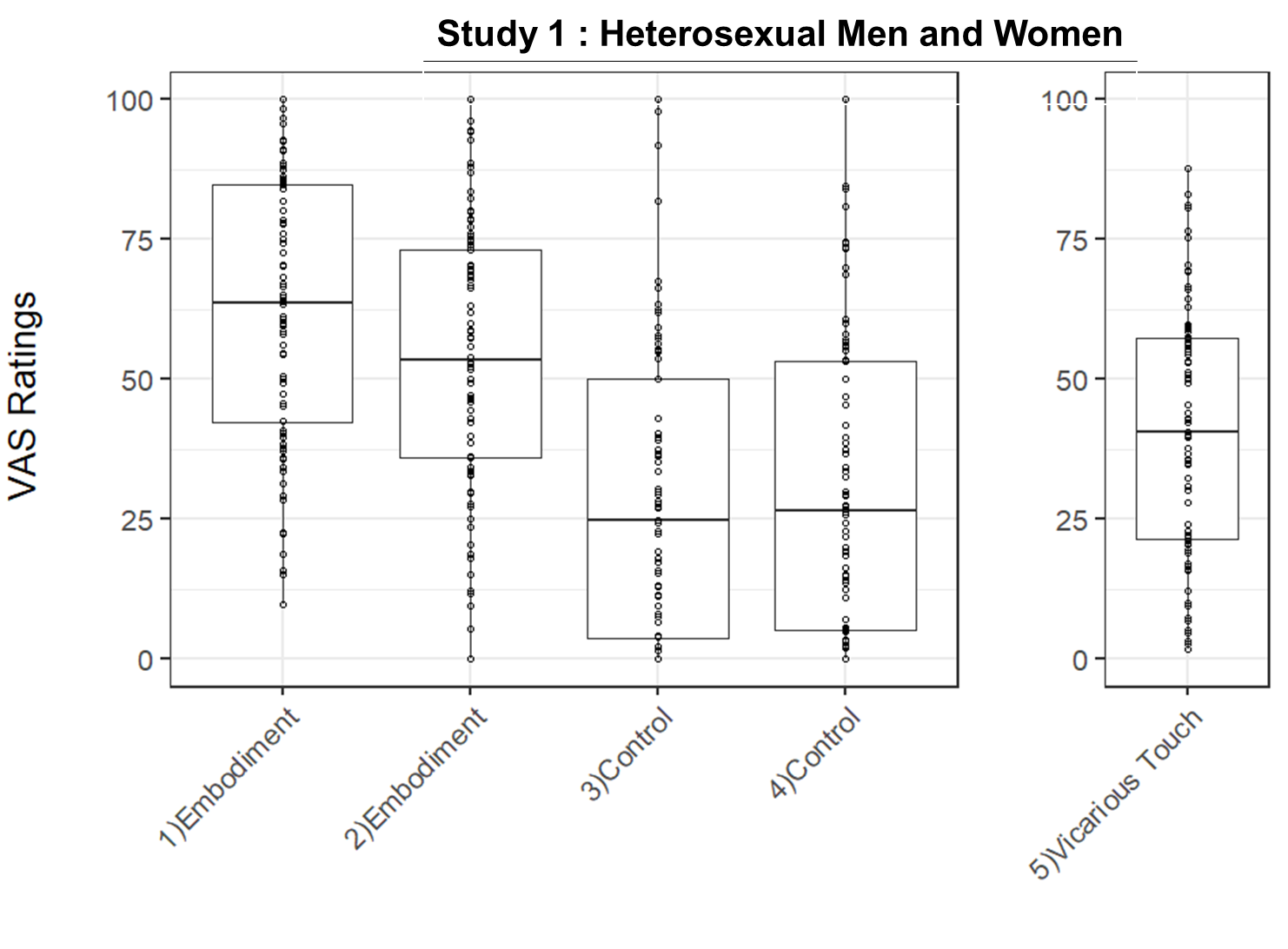


**Figure S2.** Study 1 (Heterosexual men and women): Boxplot of the ratings for each item of the embodiment questionnaire (listed above). On each box, the central mark indicates the median, and the lower and upper hinges correspond to the 25^th^ and 75^th^ percentiles. The upper whisker extends to the largest value no further than 1.5 * IQR (inter-quartile range) from the hinge. The lower whisker extends to the smallest value at most 1.5 * IQR of the hinge. Dots represent individual data points.

| **STUDY 1 : Heterosexual Men and Women** | | | | | | |
| --- | --- | --- | --- | --- | --- | --- |
| **Appropriateness** | | | | | | |
| **Fixed effect** | **Est.** | **S.E.** | ***t*** | ***χ^2^*** | **df** | ***P*** |
| (Intercept) | 40.227 | 3.123 | 12.881 |  |  |  |
| Gender | -16.421 | 4.417 | -3.718 | 1.5913 | 1 | 0.207142 |
| TouchingAvatar | -14.040 | 2.124 | -6.609 | 16.7644 | 1 | 4.232e-05*** |
| Area | 6.719 | 2.993 | 2.245 | 148.2278 | 2 | <2.2e-16*** |
| Gender:TouchingAvatar | 14.186 | 3.004 | 4.722 | 29.7135 | 1 | 5.009e-08*** |
| Gender:Area | 16.539 | 4.233 | 3.907 | 16.6781 | 2 | 0.000239*** |
| TouchingAvatar:Area | 10.142 | 3.359 | 3.020 | 11.1576 | 2 | 0.003777** |
| Gender:TouchingAvatar:Area | -4.518 | 4.750 | -0.951 | 2.6823 | 2 | 0.261544 |
| **Erogeneity** | | | | | | |
| **Fixed Effect** | **Est.** | **S.E.** | ***t*** | ***χ^2^*** | **df** | ***P*** |
| (Intercept) | 54.0820 | 4.8060 | 11.253 |  |  |  |
| Gender | -18.8773 | 6.7967 | -2.777 | 0.0100 | 1 | 0.920306 |
| TouchingAvatar | -28.7878 | 2.0472 | -14.062 | 10.6027 | 1 | 0.001129** |
| Area | -18.1784 | 4.3265 | -4.202 | 59.6825 | 2 | 1.097e-13*** |
| Gender:TouchingAvatar | 47.6674 | 2.8952 | 16.464 | 332.5051 | 1 | <2.2e-16*** |
| Gender:Area | 0.1154 | 6.1185 | 0.019 | 3.5679 | 2 | 0.167975 |
| TouchingAvatar:Area | 10.4104 | 3.2370 | 3.216 | 6.0112 | 2 | 0.049510* |
| Gender:TouchingAvatar:Area | -18.6943 | 4.5778 | -4.084 | 38.7192 | 2 | 3.910e-09*** |
| **Pleasantness** | | | | | | |
| **Fixed effect** | **Est.** | **S.E.** | ***t*** | ***χ^2^*** | **df** | ***P*** |
| (Intercept) | 59.916 | 2.934 | 20.418 |  |  |  |
| Gender | -24.617 | 4.150 | -5.932 | 1.1062 | 1 | 0.292903 |
| TouchingAvatar | -23.710 | 1.884 | -12.583 | 35.3689 | 1 | 2.728e-09*** |
| Area | -8.388 | 2.876 | -2.917 | 34.1384 | 2 | 3.863e-08*** |
| Gender:TouchingAvatar | 30.466 | 2.665 | 11.433 | 134.6086 | 1 | <2.2e-16*** |
| Gender:Area | 19.911 | 4.067 | 4.896 | 29.0868 | 2 | 4.829e-07*** |
| TouchingAvatar:Area | 12.622 | 2.979 | 4.237 | 10.6670 | 2 | 0.004827** |
| Gender:TouchingAvatar:Area | -13.731 | 4.213 | -3.259 | 29.0298 | 2 | 4.969e-07*** |
| **Arousal** | | | | | | |
| **Fixed effect** | **Est.** | **S.E.** | ***t*** | ***χ^2^*** | **df** | ***P*** |
| (Intercept) | 62.183488 | 3.094952 | 20.092 |  |  |  |
| Gender | -1.184963 | 4.376923 | -0.271 | 4.6854 | 1 | 0.0304192* |
| TouchingAvatar | -10.318892 | 1.942187 | -5.313 | 11.5759 | 1 | 0.0006681*** |
| Area | -7.495625 | 3.508645 | -2.136 | 11.4548 | 2 | 0.0032555** |
| Gender:TouchingAvatar | 17.656611 | 2.746667 | 6.428 | 46.7853 | 1 | 7.92e-12*** |
| Gender:Area | 6.290730 | 4.961974 | 1.268 | 0.1788 | 2 | 0.9144793 |
| TouchingAvatar:Area | 3.894908 | 3.070867 | 1.268 | 10.0490 | 2 | 0.0065750** |
| Gender:TouchingAvatar:Area | -12.101570 | 4.342862 | -2.787 | 7.9714 | 2 | 0.0185793* |
| **SCR** | | | | | | |
| **Fixed effect** | **Est.** | **S.E.** | ***t*** | ***χ^2^*** | **df** | ***P*** |
| (Intercept) | 0.81179 | 0.08411 | 9.651 |  |  |  |
| Gender | -0.26718 | 0.11895 | -2.246 | 3.090 | 1 | 0.078734 |
| TouchingAvatar | -0.01141 | 0.06372 | -0.179 | 5.298 | 1 | 0.021343* |
| Area | -0.20894 | 0.05088 | -4.107 | 5.599 | 2 | 0.060833 |
| Gender:TouchingAvatar | 0.06478 | 0.09011 | 0.719 | 0.544 | 1 | 0.460464 |
| Gender:Area | 0.13905 | 0.07195 | 1.933 | 6.608 | 2 | 0.036733* |
| TouchingAvatar:Area | 0.19567 | 0.07195 | 2.720 | 13.611 | 2 | 0.001108*** |
| Gender:TouchingAvatar:Area | -0.02 | 0.10175 | -0.099 | 0.041 | 2 | 0.979340 |
| **HR** | | | | | | |
| **Fixed effect** | **Est.** | **S.E.** | ***t*** | ***χ^2^*** | **df** | ***P*** |
| (Intercept) | 7.365079 | 0.246053 | 29.933 |  |  |  |
| Gender | 0.611111 | 0.347971 | 1.756 | 3.3239 | 1 | 0.06828 |
| TouchingAvatar | -0.031746 | 0.105660 | -0.300 | 1.5014 | 1 | 0.22045 |
| Area | -0.019841 | 0.076184 | -0.260 | 2.2829 | 2 | 0.31935 |
| Gender:TouchingAvatar | -0.182540 | 0.149426 | -1.222 | 0.8326 | 1 | 0.36152 |
| Gender:Area | 0.019841 | 0.107741 | 0.184 | 5.3875 | 2 | 0.06763 |
| TouchingAvatar:Area | -0.051587 | 0.107741 | -0.479 | 1.4806 | 2 | 0.47697 |
| Gender:TouchingAvatar:Area | 0.230159 | 0.152368 | 1.511 | 2.7945 | 2 | 0.24727 |
| **Ownership** | | | | | | |
| **Fixed effect** | **Est.** | **S.E.** | ***t*** | ***χ^2^*** | **df** | ***P*** |
| (Intercept) | 27.612 | 4.996 | 5.526 |  |  |  |
| Gender | 2.526 | 7.066 | 0.358 | 0.0142 | 1 | 0.90531 |
| TouchingAvatar | 4.771 | 3.703 | 1.288 | 0.1215 | 1 | 0.72738 |
| Question | 34.224 | 6.866 | 4.985 | 38.6522 | 1 | 5.064e-10*** |
| Gender:TouchingAvatar | -3.796 | 5.237 | -0.725 | 0.0194 | 1 | 0.88910 |
| Gender: Question | -5.611 | 9.709 | -0.578 | 0.0675 | 1 | 0.79508 |
| TouchingAvatar: Question | -10.316 | 5.237 | -1.970 | 3.6107 | 1 | 0.05741 |
| Gender:TouchingAvatar: Question | 6.559 | 7.406 | 0.886 | 0.7843 | 1 | 0.37582 |
| **Vicarious Touch** | | | | | | |
| **Fixed effect** | **Est.** | **S.E.** | ***t*** | ***χ^2^*** | **df** | ***p*** |
| (Intercept) | 40.795 | 4.636 | 8.800 |  |  |  |
| Gender | 1.785 | 6.556 | 0.272 | 1.0079 | 1 | 0.31540 |
| TouchingAvatar | -5.600 | 3.556 | -1.575 | 0.2701 | 1 | 0.60329 |
| Gender:TouchingAvatar | 8.587 | 5.029 | 1.707 | 2.9152 | 1 | 0.08775 |

**Table S2**. Study 1: Summary of the fixed effects for each model. Est: estimate. S.E.: standard error. Df : degrees of freedom. Signif.codes: '***' 0.001 '**' 0.01 '*' 0.05 '*’.

**Appropriateness**

In the table below, we show the estimated marginal means for the three 2-level significant interactions found in the analysis of the Appropriateness ratings.

| **Study 1 : Heterosexual Men and Women** | | | | | | |
| --- | --- | --- | --- | --- | --- | --- |
| **Participants’**  **Gender** | **Touching_Avatar** | **Mean** | **SE** | **df** | **lower.CL** | **upper.CL** |
| Men | Female | 49 | 2.93 | 50 | 43.1 | 54.8 |
| Women | Female | 44.4 | 2.93 | 50 | 38.5 | 50.3 |
| Men | Male | 40.3 | 2.93 | 50 | 34.5 | 46.2 |
| Women | Male | 45.9 | 2.93 | 50 | 40 | 51.8 |
| **Participants’**  **Gender** | **Area** | **Mean** | **SE** | **df** | **lower.CL** | **upper.CL** |
| Men | Intimate | 33.2 | 3.01 | 44.1 | 27.1 | 39.3 |
| Women | Intimate | 23.9 | 3.01 | 44.1 | 17.8 | 29.9 |
| Men | Neutral | 45 | 3.42 | 44.1 | 38.1 | 51.9 |
| Women | Neutral | 49.9 | 3.42 | 44.1 | 43.1 | 56.8 |
| Men | Social | 55.7 | 3.68 | 44.1 | 48.3 | 63.2 |
| Women | Social | 61.6 | 3.68 | 44.1 | 54.2 | 69 |
| **Touching_Avatar** | **Area** | **Mean** | **SE** | **df** | **lower.CL** | **upper.CL** |
| Female | Intimate | 32 | 2.26 | 56.4 | 27.5 | 36.5 |
| Male | Intimate | 25.1 | 2.26 | 56.4 | 20.5 | 29.6 |
| Female | Neutral | 47 | 2.59 | 58.5 | 41.8 | 52.2 |
| Male | Neutral | 47.9 | 2.59 | 58.5 | 42.8 | 53.1 |
| Female | Social | 61 | 2.76 | 56.4 | 55.5 | 66.5 |
| Male | Social | 56.4 | 2.76 | 56.4 | 50.8 | 61.9 |

**Table S3.** Study 1 (Heterosexual men and women): means, SE, df, upper and lower CL for: the interactions Gender:TouchingAvatar, Gender:Area and TouchingAvatar:Area found in the analysis of the Appropriateness ratings.

**Erogeneity**

In the table below, we show the estimated marginal means for the significant three-level interaction found in the analysis of the Erogeneity ratings.

| **Study 1 : Heterosexual Men and Women** | | | | | | | |
| --- | --- | --- | --- | --- | --- | --- | --- |
| **Touching_Avatar** | **Area** | **Gender** | **mean** | **SE** | **df** | **lower.CL** | **upper.CL** |
| Female | Intimate | Men | 54.1 | 4.92 | 48.4 | 44.19 | 64 |
| Male | Intimate | Men | 25.3 | 4.92 | 48.4 | 15.4 | 35.2 |
| Female | Neutral | Men | 35.9 | 4.12 | 54 | 27.64 | 44.2 |
| Male | Neutral | Men | 17.5 | 4.12 | 54 | 9.27 | 25.8 |
| Female | Social | Men | 22.7 | 3.78 | 56.2 | 15.13 | 30.3 |
| Male | Social | Men | 13 | 3.78 | 56.2 | 5.4 | 20.6 |
| Female | Intimate | Women | 35.2 | 4.92 | 48.4 | 25.31 | 45.1 |
| Male | Intimate | Women | 54.1 | 4.92 | 48.4 | 44.19 | 64 |
| Female | Neutral | Women | 17.1 | 4.12 | 54 | 8.88 | 25.4 |
| Male | Neutral | Women | 27.7 | 4.12 | 54 | 19.48 | 36 |
| Female | Social | Women | 11.9 | 3.78 | 56.2 | 4.35 | 19.5 |
| Male | Social | Women | 22.7 | 3.78 | 56.2 | 15.11 | 30.3 |

**Table S4.** Study 1 (Heterosexual men and women): means, SE, df, upper and lower CL for the interaction TouchingAvatar:Gender:Area found in the analysis of the Erogeneity ratings.

**(Un)pleasantness**

The linear mixed effects model (R^2^_marginal_ = 0.178, R^2^_conditional_ =0.485) revealed a main effect of the Touching avatar and of the Area touched. Moreover, three 2-way interactions and one triple interaction were significant (Fig S3).The interaction between Gender, Touching avatar and area (χ2(2)=29.02, p<.001) (Table.S2,Table.S5 and FigureS3) showed that men considered as more pleasant touches from a female avatar-compared to the male one- on intimate (p<.001) and neutral (p<.001) but equally (un)pleasant on the social area (p=.112); women rated as more pleasant touches on intimate (p=.01) from a male avatar and equally (un)pleasant touches from male and female avatars on neutral (p=0.38) and social (p=1) areas. Moreover, men touched by a female avatar rated the three areas as equally (un)pleasant (intimate vs neutral p=.17; intimate vs social p=.50; social vs neutral p=1). In heterosexual men, similarly to when they were touched by the female avatar, all the areas touched were considered equally (un)pleasant when touched by the male avatar (intimate vs neutral p=.95; intimate vs social p=.09, social vs neutral p=.86). Differently, when heterosexual women were touched by the male avatar there were no differences between social and neutral areas (p=.47) but touches on social and neutral were considered more pleasurable than on intimate areas (p<.001, p=.029, respectively). When they were touched by the female avatar, heterosexual women considered touches as more pleasant on neutral and social than intimate (p=.009, p<.001, respectively) and more pleasant on social than neutral (p=.02).

| **Study 1 : Heterosexual Men and Women** | | | | | | | |
| --- | --- | --- | --- | --- | --- | --- | --- |
| **Touching_Avatar** | **Area** | **Gender** | **mean** | **SE** | **df** | **lower.CL** | **upper.CL** |
| Female | Intimate | Men | 59.9 | 3 | 54.8 | 53.9 | 65.9 |
| Male | Intimate | Men | 36.2 | 3 | 54.8 | 30.2 | 42.2 |
| Female | Neutral | Men | 51.5 | 3.2 | 59 | 45.1 | 57.9 |
| Male | Neutral | Men | 40.4 | 3.2 | 59 | 34 | 46.8 |
| Female | Social | Men | 52.9 | 2.8 | 65 | 47.3 | 58.5 |
| Male | Social | Men | 46 | 2.8 | 65 | 40.4 | 51.6 |
| Female | Intimate | Women | 35.3 | 3 | 54 | 29.3 | 41.3 |
| Male | Intimate | Women | 42.1 | 3 | 54 | 36 | 48.1 |
| Female | Neutral | Women | 46.8 | 3.2 | 59 | 40.4 | 53.2 |
| Male | Neutral | Women | 52.5 | 3.2 | 59 | 46.1 | 58.9 |
| Female | Social | Women | 58.5 | 2.8 | 65 | 52.9 | 64.1 |

**Table S5.** Study 1 (Heterosexual men and women): means, SE, df, upper and lower CL for the interaction Gender:TouchingAvatar:area of (Un)pleasantness


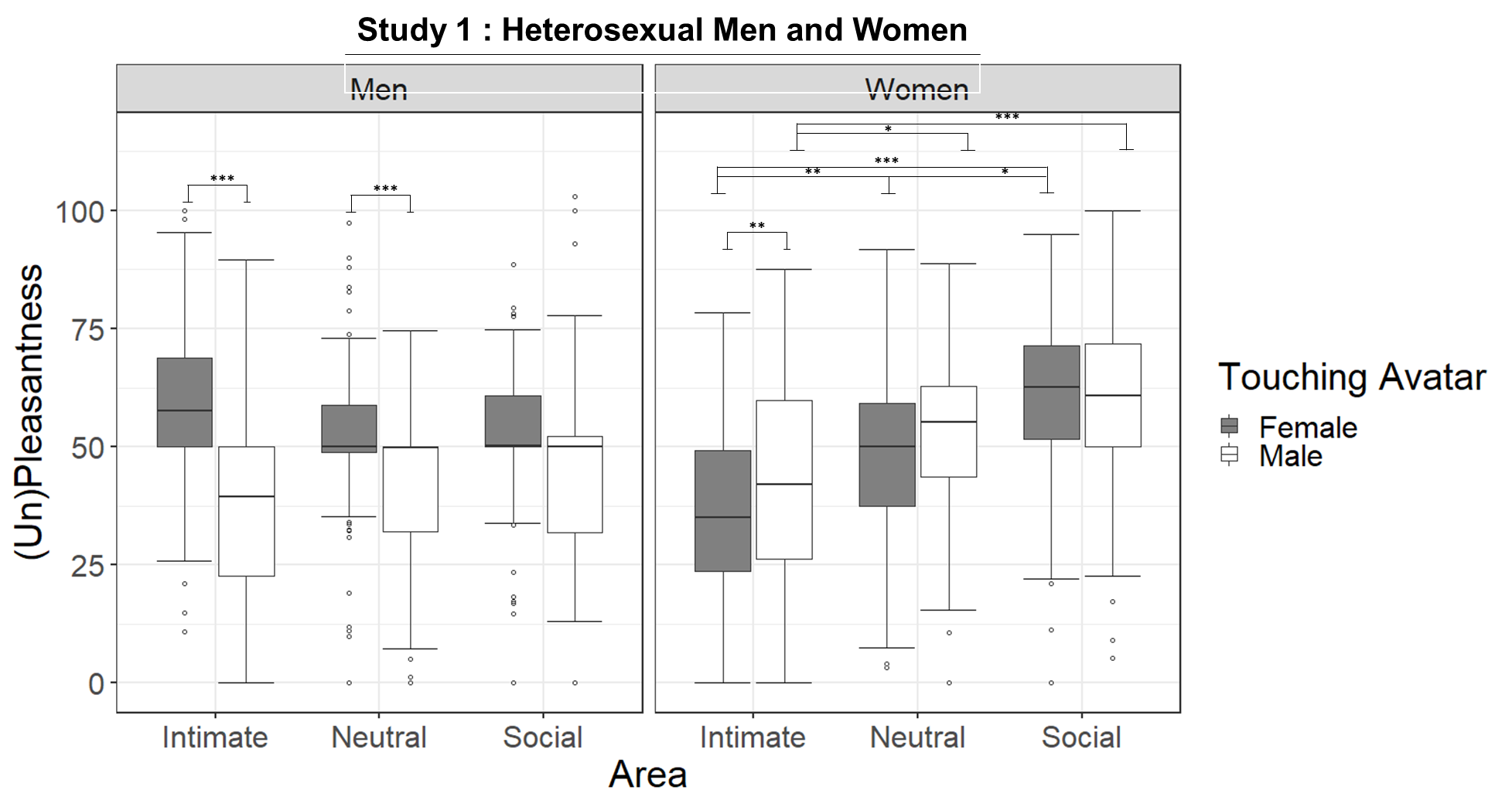


**Figure S3.** Study 1 (Heterosexual men and women)**:** Boxplots of the (Un)Pleasantness ratings for the interaction between Gender, Touching Avatar and Area. On each box, the central mark indicates the median, and the lower and upper hinges correspond to the 25th and 75th percentiles. The upper whisker extends to the largest value no further than 1.5 * IQR (inter-quartile range) from the hinge. The lower whisker extends to the smallest value at most 1.5 * IQR of the hinge. Points outside this range are shown individually.

**Arousal**

The linear mixed effects model (R^2^_marginal_ = 0.092, R^2^_conditional_ =0.493) revealed a main effect of the Gender, the Touching avatar and of the Area touched along with two 2-way interactions and one triple interaction (see Table.S3). The interaction between Gender, Touching avatar and Area (χ2(2)=7.97, p=.01) (TableS6, FigureS4) showed that when heterosexual men were touched by a female avatar on intimate and neutral areas it was more arousing compared to the touch from the male avatar (p<.001, p=0.001, respectively) with no differences on the social area were found (p=0.23). For heterosexual women, only touches on the intimate areas were more arousing when delivered by a male touching avatar (p=.009) and no differences for neutral(p=.69) and social (p=74). No differences were found between the areas for heterosexual men receiving touches from the female touching avatar or the male one; for heterosexual women only the touch on the intimate area from the male touching avatar was more arousing compared to the neutral area (p=.019) but not to the social one (p=.85)while no differences between these latter two (p=.66) were found. Lastly. when heterosexual women were touched by a female avatar no differences were detected between the areas.

| **Study 1 : Heterosexual Men and Women** | | | | | | | |
| --- | --- | --- | --- | --- | --- | --- | --- |
| **Touching_Avatar** | **Area** | **Gender** | **mean** | **SE** | **Df** | **lower.CL** | **upper.CL** |
| Female | Intimate | Men | 62.2 | 3.16 | 54.2 | 55.8 | 68.5 |
| Male | Intimate | Men | 51.9 | 3.16 | 54.2 | 44.5 | 58.2 |
| Female | Neutral | Men | 54.7 | 3.55 | 56.6 | 47.6 | 61.8 |
| Male | Neutral | Men | 44.4 | 3.55 | 56.6 | 37.3 | 51.5 |
| Female | Social | Men | 53.6 | 3.75 | 55 | 46.1 | 61.2 |
| Male | Social | Men | 47.2 | 3.75 | 55 | 39.7 | 54.7 |
| Female | Intimate | Women | 61 | 3.13 | 54.2 | 54.7 | 67.3 |
| Male | Intimate | Women | 68.3 | 3.13 | 54.2 | 62 | 74.7 |
| Female | Neutral | Women | 59.8 | 3.55 | 56.6 | 52.7 | 66.9 |
| Male | Neutral | Women | 55 | 3.55 | 56.6 | 7.9 | 62.1 |
| Female | Social | Women | 57.5 | 3.75 | 55 | 50 | 65 |
| Male | Social | Women | 62.1 | 3.75 | 55 | 54.6 | 69.6 |

**Table S6.** means, SE, df, upper and lower CL for the interaction Gender:TouchingAvatar:Area of Arousal


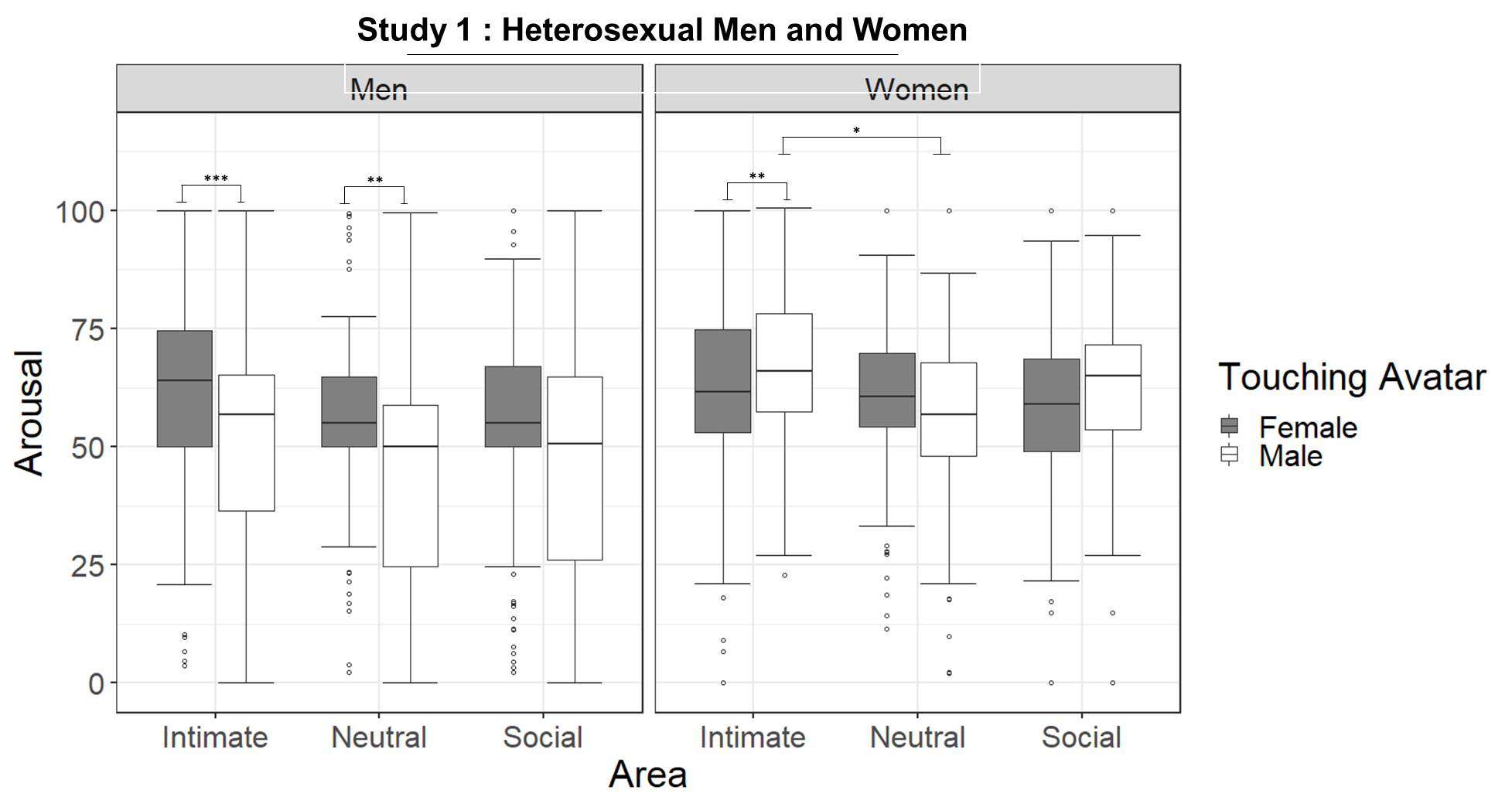


**Figure. S4.** Study 1 (Heterosexual men and women): Boxplots of the Arousal ratings for the interaction between Gender, Touching Avatar and Area. On each box, the central mark indicates the median, and the lower and upper hinges correspond to the 25th and 75th percentiles. The upper whisker extends to the largest value no further than 1.5 * IQR (inter-quartile range) from the hinge. The lower whisker extends to the smallest value at most 1.5 * IQR of the hinge. Points outside this range are shown individually. Signif.codes: '***' 0.001 '**' 0.01 '*' 0.05 '*’.

**SCR**.

In the table below, we show the estimated marginal means for the two 2-level significant interactions found in the analysis of the SCR ratings.

| **Study 1 : Heterosexual Men and Women** | | | | | | |
| --- | --- | --- | --- | --- | --- | --- |
| **Gender** | **Area** | **Mean** | **SE** | **df** | **lower.CL** | **upper.CL** |
| Men | Intimate | 0.806 | 0.08 | 48.0 | 0.631 | 0.981 |
| Women | intimate | 0.571 | 0.08 | 48.0 | 0.396 | 0.746 |
| Men | Neutral | 0.695 | 0.08 | 51.4 | 0.517 | 0.873 |
| Women | Neutral | 0.589 | 0.08 | 51.4 | 0.411 | 0.766 |
| Men | Social | 0.716 | 0.08 | 51.4 | 0.539 | 0.894 |
| Women | Social | 0.554 | 0.08 | 51.4 | 0.377 | 0.732 |
| **Touching avatar** | **Area** | **Mean** | **SE** | **Df** | **lower.CL** | **upper.CL** |
| Female | Intimate | 0.678 | 0.06 | 52.5 | 0.556 | 0.800 |
| Male | Intimate | 0.699 | 0.06 | 50.2 | 0.558 | 0.840 |
| Female | Neutral | 0.539 | 0.06 | 60.4 | 0.413 | 0.665 |
| Male | Neutral | 0.745 | 0.07 | 55.9 | 0.601 | 0.889 |
| Female | Social | 0.601 | 0.06 | 60.4 | 0.475 | 0.727 |
| Male | Social | 0.669 | 0.07 | 55.9 | 0.525 | 0.813 |

**Table S7.** Study 1 (Heterosexual men and women): means, SE, df, upper and lower CL for the interaction Gender: Area and TouchingAvatar:Area found in the analysis of the SCR.

**HR**.

No significant difference was found. In the table below we show the estimated marginal means and confidence intervals for all the combinations between the factors’ levels.

| **Study 1 : Heterosexual Men and Women** | | | | | | | |
| --- | --- | --- | --- | --- | --- | --- | --- |
| **Touching_Avatar** | **Area** | **Gender** | **mean** | **SE** | **df** | **lower.CL** | **upper.CL** |
| Female | Intimate | Men | 7.37 | 0.252 | 46.1 | 6.86 | 7.87 |
| Male | Intimate | Men | 7.33 | 0.257 | 46.0 | 6.82 | 7.85 |
| Female | Neutral | Men | 7.35 | 0.254 | 47.9 | 6.83 | 7.86 |
| Male | Neutral | Men | 7.26 | 0.260 | 47.7 | 6.74 | 7.78 |
| Female | Social | Men | 7.30 | 0.254 | 47.9 | 6.79 | 7.81 |
| Male | Social | Men | 7.36 | 0.260 | 47.7 | 6.84 | 7.88 |
| Female | Intimate | Women | 7.98 | 0.252 | 46.1 | 7.47 | 8.48 |
| Male | Intimate | Women | 7.76 | 0.257 | 46.0 | 7.24 | 8.28 |
| Female | Neutral | Women | 7.98 | 0.254 | 47.9 | 7.46 | 8.49 |
| Male | Neutral | Women | 7.94 | 0.260 | 47.7 | 7.42 | 8.46 |
| Female | Social | Women | 8.07 | 0.254 | 47.9 | 7.56 | 8.58 |
| Male | Social | Women | 7.94 | 0.260 | 47.7 | 7.42 | 8.46 |

**Table S8**. Study 1: HR (the number of heartbeats in a 6-second post-stimulus time window) means, SE, df, upper and lower CL for all the combinations between the factors’ levels.

**Study 2 : Gay men and Lesbian women**

**Mixed Effects Model Syntax**

**Appropriateness:**

*lmer(Appropriateness ~ Gender* TouchingAvatar * Area+ (1 +Area| Participant))*

**Erogeneity:**

*lmer(Erogeneity ~ Gender* TouchingAvatar * Area+ (1 +Area| Participant))*

**SCR:**

*lmer(SCR ~ Gender* TouchingAvatar * Area+ (1 +TouchingAvatar| Participant))*

**Ownership:**

*lmer(Rating ~ Gender* TouchingAvatar * Question+ (1 +Question| Participant))*

**Vicarious Touch:**

*lmer(VicariousTouch ~ Gender* TouchingAvatar+ (1| Participant))*

**UnPleasantness:**

*lmer(UnPleasantness: ~ Gender* TouchingAvatar * Area+ (1 +Area| Participant))*

**Arousal:**

*lmer(Arousal: ~ Gender* TouchingAvatar * Area+ (1 +Area| Participant))*

**HR:**

*lmer(HR ~ Gender* TouchingAvatar * Area+ (1 +TouchingAvatar| Participant))*


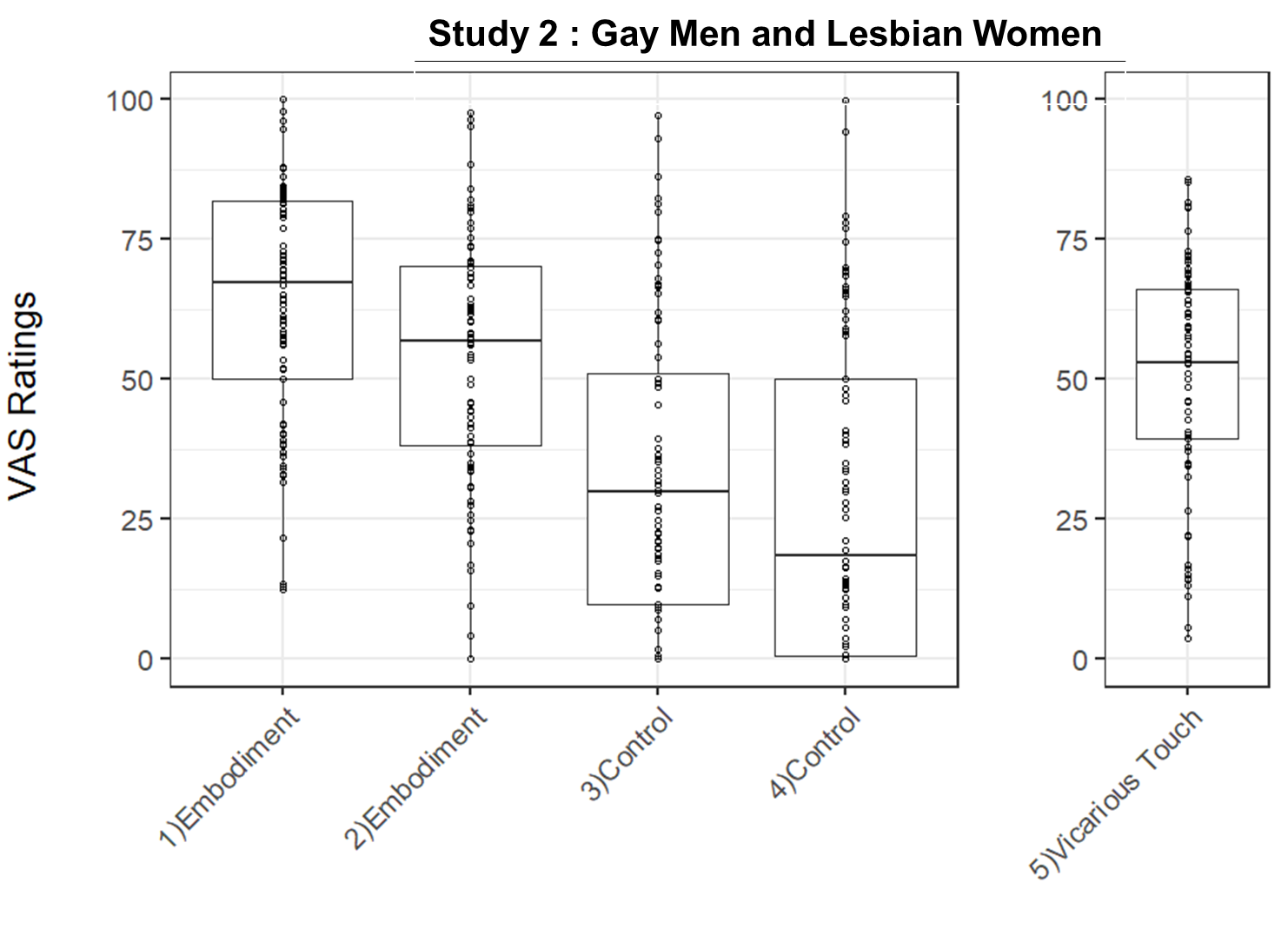


**Figure. S5**. Study 2 (Gay men and Lesbian women): Boxplot of the ratings for each item of the embodiment questionnaire (listed in Table.S9). On each box, the central mark indicates the median, and the lower and upper hinges correspond to the 25^th^ and 75^th^ percentiles. The upper whisker extends to the largest value no further than 1.5 * IQR (inter-quartile range) from the hinge. The lower whisker extends to the smallest value at most 1.5 * IQR of the hinge. Dots represent individual data points

| **STUDY 2: Gay men and Lesbian women** | | | | | | |
| --- | --- | --- | --- | --- | --- | --- |
| **Appropriateness** | | | | | | |
| **Fixed effect** | **Est.** | **S.E.** | ***t*** | ***χ^2^*** | **df** | ***p*** |
| (Intercept) | 37.955 | 4.941 | 7.682 |  |  |  |
| Gender | 1.638 | 6.988 | 0.234 | 0.1921 | 1 | 0.66121 |
| TouchingAvatar | 7.774 | 2.458 | 3.162 | 5.3116 | 1 | 0.02118* |
| Area | 9.644 | 4.361 | 2.212 | 44.3584 | 2 | 2.332e-10*** |
| Gender:TouchingAvatar | -23.388 | 3.473 | -6.734 | 47.5507 | 1 | 5.360e-12*** |
| Gender:Area | 8.438 | 6.282 | 1.343 | 8.2662 | 2 | 0.01603* |
| TouchingAvatar:Area | -3.597 | 3.882 | -0.927 | 1.0832 | 2 | 0.58182 |
| Gender:TouchingAvatar:Area | 12.618 | 5.488 | 2.299 | 8.7227 | 2 | 0.01276* |
| **Erogeneity** | | | | | | |
| **Fixed effect** | **Est.** | **S.E.** | ***t*** | ***χ^2^*** | **df** | ***p*** |
| (Intercept) | 32.026 | 4.359 | 7.347 |  |  |  |
| Gender | 23.312 | 6.165 | 3.782 | 2.2678 | 1 | 0.1320846 |
| TouchingAvatar | 32.560 | 2.326 | 13.996 | 37.8496 | 1 | 7.641e-10*** |
| Area | -8.632 | 3.687 | -2.341 | 94.8214 | 2 | <2.2e-16*** |
| Gender:TouchingAvatar | -38.572 | 3.286 | -11.736 | 180.6712 | 1 | <2.2e-16*** |
| Gender:Area | -8.121 | 5.214 | -1.558 | 0.6551 | 2 | 0.7206714 |
| TouchingAvatar:Area | -21.259 | 3.674 | -5.787 | 29.4045 | 2 | 4.120e-07*** |
| Gender:TouchingAvatar:Area | 21.979 | 5.193 | 4.232 | 18.3189 | 2 | 0.0001052*** |
| **Pleasantness** | | | | | | |
| **Fixed effect** | **Est.** | **S.E.** | ***t*** | ***χ^2^*** | **df** | ***p*** |
| (Intercept) | 47.9160 | 3.3772 | 14.188 |  |  |  |
| Gender | 9.7880 | 4.7761 | 2.049 | 0.0334 | 1 | 0.85496 |
| TouchingAvatar | 19.2317 | 2.1842 | 8.805 | 0.6141 | 1 | 0.43323 |
| Area | 1.5754 | 3.5278 | 0.447 | 3.6584 | 2 | 0.16054 |
| Gender:TouchingAvatar | -32.2170 | 3.0856 | -10.441 | 102.7826 | 1 | <2.2e-16*** |
| Gender:Area | -5.9351 | 4.9891 | -1.190 | 8.9977 | 2 | 0.01112* |
| TouchingAvatar:Area | -13.5232 | 3.4490 | -3.921 | 4.4014 | 2 | 0.11073 |
| Gender:TouchingAvatar:Area | 20.7938 | 4.8756 | 4.265 | 25.3654 | 2 | 3.104e-06*** |
| **Arousal** | | | | | | |
| **Fixed effect** | **Est.** | **S.E.** | ***t*** | ***χ^2^*** | **df** | ***p*** |
| (Intercept) | 57.6040 | 3.4801 | 16.552 |  |  |  |
| Gender | 8.2450 | 4.9216 | 1.675 | 0.0306 | 1 | 0.86120 |
| TouchingAvatar | 10.4995 | 1.9783 | 5.307 | 6.0639 | 1 | 0.01380* |
| Area | -6.6456 | 3.4485 | -1.927 | 23.1250 | 2 | 9.516e-06*** |
| Gender:TouchingAvatar | -11.8345 | 2.7948 | -4.235 | 37.2641 | 1 | 1.032e-09*** |
| Gender:Area | -2.5959 | 4.8770 | -0.532 | 0.5554 | 2 | 0.75751 |
| TouchingAvatar:Area | -4.8796 | 3.1240 | -1.562 | 4.8527 | 2 | 0.08836 |
| Gender:TouchingAvatar:Area | 1.3609 | 4.4161 | 0.308 | 0.1071 | 2 | 0.94784 |
| **SCR** | | | | | | |
| **Fixed effect** | **Est.** | **S.E.** | ***t*** | ***χ^2^*** | **df** | ***p*** |
| (Intercept) | 0.94738 | 0.08844 | 10.712 |  |  |  |
| Gender | -0.10756 | 0.12507 | -0.860 | 0.3779 | 1 | 0.538713 |
| TouchingAvatar | -0.16825 | 0.06984 | -2.409 | 0.5679 | 1 | 0.451094 |
| Area | -0.25042 | 0.05322 | -4.705 | 11.6074 | 2 | 0.003016** |
| Gender:TouchingAvatar | 0.07337 | 0.09877 | 0.743 | 0.0447 | 1 | 0.832515 |
| Gender:Area | 0.12946 | 0.07526 | 1.720 | 2.4598 | 2 | 0.292318 |
| TouchingAvatar:Area | 0.31063 | 0.07526 | 4.127 | 19.2893 | 2 | 6.477e-05*** |
| Gender:TouchingAvatar:Area | -0.15663 | 0.10644 | -1.472 | 2.2320 | 2 | 0.327588 |
| **HR** | | | | | | |
| **Fixed effect** | **Est.** | **S.E.** | ***t*** | ***χ^2^*** | **df** | ***p*** |
| (Intercept) | 8.07937 | 0.25571 | 31.596 |  |  |  |
| Gender | -0.18770 | 0.36612 | -0.513 | 0.3742 | 1 | 0.540731 |
| TouchingAvatar | -0.05556 | 0.13618 | -0.408 | 1.0576 | 1 | 0.303770 |
| Area | -0.11508 | 0.07834 | -1.469 | 0.5529 | 2 | 0.758464 |
| Gender:TouchingAvatar | -0.12778 | 0.19498 | -0.655 | 0.2223 | 1 | 0.637282 |
| Gender:Area | -0.01409 | 0.11216 | -0.126 | 1.5908 | 2 | 0.451402 |
| TouchingAvatar:Area | 0.12698 | 0.11079 | 1.146 | 10.8828 | 2 | 0.004333** |
| Gender:TouchingAvatar:Area | 0.11885 | 0.15863 | 0.749 | 0.5729 | 2 | 0.750934 |
| **Ownership** | | | | | | |
| **Fixed effect** | **Est.** | **S.E.** | ***t*** | ***χ^2^*** | **df** | ***p*** |
| (Intercept) | 29.262 | 5.273 | 5.549 |  |  |  |
| Gender | 4.484 | 7.458 | 0.601 | 0.0434 | 1 | 0.8349 |
| TouchingAvatar | -4.119 | 3.379 | -1.219 | 0.0048 | 1 | 0.9445 |
| Question | 32.590 | 7.298 | 4.465 | 32.6544 | 1 | 1.101e-08*** |
| Gender:TouchingAvatar | 5.946 | 4.779 | 1.244 | 1.0788 | 1 | 0.2990 |
| Gender:Question | -11.507 | 10.321 | -1.115 | 2.0444 | 1 | 0.1528 |
| TouchingAvatar:Question | 4.493 | 4.779 | 0.940 | 0.3704 | 1 | 0.5428 |
| Gender: TouchingAvatar: Question | -4.873 | 6.759 | -0.721 | 0.5199 | 1 | 0.4709 |
| **Vicarious Touch** | | | | | | |
| **Fixed effect** | **Est.** | **S.E.** | ***t*** | ***χ^2^*** | **df** | ***p*** |
| (Intercept) | 52.149 | 4.714 | 11.064 |  |  |  |
| Gender | -4.508 | 6.607 | -0.682 | 1.7400 | 1 | 0.1871 |
| TouchingAvatar | 3.147 | 2.312 | 1.361 | 0.1792 | 1 | 0.6721 |
| Gender:TouchingAvatar | -7.861 | 3.306 | -2.378 | 5.6551 | 1 | 0.0174* |

**Table S9.** Study 2 (Gay men and Lesbian women): Summary of the fixed effects for each model. Est: estimate. S.E.: standard error. Df: degrees of freedom. Signif.codes: '***' 0.001 '**' 0.01 '*' 0.05 '*’.

**Appropriateness**

In the table below, we show the estimated marginal means for the significant three-level interaction between Touching Avatar, Area and Gender found in the analysis of the Appropriateness ratings.

| **Study 2 : Gay Men and Lesbian Women** | | | | | | | |
| --- | --- | --- | --- | --- | --- | --- | --- |
| **Touching_Avatar** | **Area** | **Gender** | **mean** | **SE** | **df** | **lower.CL** | **upper.CL** |
| Female | Intimate | Men | 37 | 5.06 | 50.1 | 27.8 | 48.1 |
| Male | Intimate | Men | 45.7 | 5.06 | 50.1 | 35.6 | 55.9 |
| Female | Neutral | Men | 47.6 | 4.54 | 56.2 | 38.5 | 56.7 |
| Male | Neutral | Men | 51.8 | 4.54 | 56.2 | 42.7 | 60.9 |
| Female | Social | Men | 51.6 | 3.86 | 62.1 | 43.9 | 59.3 |
| Male | Social | Men | 54.1 | 3.86 | 62.1 | 46.4 | 61.8 |
| Female | Intimate | Women | 39.6 | 5.06 | 50.1 | 29.4 | 49.7 |
| Male | Intimate | Women | 24 | 5.06 | 50.1 | 13.8 | 34.1 |
| Female | Neutral | Women | 47.9 | 4.54 | 56.2 | 38.9 | 57 |
| Male | Neutral | Women | 41.4 | 4.54 | 56.2 | 32.3 | 50.4 |
| Female | Social | Women | 61.7 | 3.86 | 62.1 | 54 | 69.4 |
| Male | Social | Women | 55.1 | 3.86 | 62.1 | 47.4 | 62.9 |

**Table S10.** Study 2 (Gay men and Lesbian women): means, SE, df, upper and lower CL of Appropriateness at the factors combinations of Touching Avatar, Area and Gender .

**Erogeneity**

In the table below, we show the estimated marginal means for the significant three-level interaction between Touching Avatar, Area and Gender found in the analysis of the Erogeneity ratings.

| **Study 2 : Gay Men and Lesbian Women** | | | | | | | |
| --- | --- | --- | --- | --- | --- | --- | --- |
| **Touching_Avatar** | **Area** | **Gender** | **mean** | **SE** | **df** | **lower.CL** | **upper.CL** |
| Female | Intimate | Men | 32 | 4.46 | 51.1 | 23.07 | 41 |
| Male | Intimate | Men | 64.6 | 4.46 | 51.1 | 55.63 | 73.5 |
| Female | Neutral | Men | 23.4 | 4.14 | 57.3 | 15.1 | 31.7 |
| Male | Neutral | Men | 34.7 | 4.14 | 57.3 | 26.4 | 43 |
| Female | Social | Men | 15.1 | 4.06 | 57.9 | 6.97 | 23.2 |
| Male | Social | Men | 28.8 | 4.06 | 57.9 | 20.65 | 36.9 |
| Female | Intimate | Women | 55.3 | 4.46 | 51.1 | 46.38 | 64.3 |
| Male | Intimate | Women | 49.3 | 4.46 | 51.1 | 40.37 | 58.3 |
| Female | Neutral | Women | 38.6 | 4.14 | 57.3 | 30.29 | 46.9 |
| Male | Neutral | Women | 33.3 | 4.14 | 57.3 | 25 | 41.6 |
| Female | Social | Women | 36.6 | 4.06 | 57.9 | 28.48 | 44.7 |
| Male | Social | Women | 23.6 | 4.06 | 57.9 | 15.43 | 31.7 |

**Table S11.** Study 2 (Gay men and Lesbian women): means, SE, df, upper and lower CL of Erogeneity at the factors combinations of Gender, Touching Avatar and Area .

**(Un)pleasantness**

The linear mixed effects model (R^2^_marginal_ = 0.089, R^2^_conditional_ =0.364) revealed two 2-way interactions and one triple interaction were found (see Table.S9).The interaction between Gender, Touching avatar and Area (χ2(2)=25.36, p<.001) (Table.S12, Figure.S6) showed that men considered as more pleasant touches from a male avatar-compared to the female one- on intimate (p<.001) but equally (un)pleasant on the social (p=.93) and neutral area (p=.599); lesbian women rated as more pleasant touches on intimate (p<.001) from a female touching avatar and equally (un)pleasant touches from male and female avatars on neutral (p=.59) and social (p=.13) areas. Moreover, for gay men touched by a female avatar, the three areas were equally (un)pleasant (intimate vs neutral p=1.00; intimate vs social p=.99; social vs neutral p=1.00). Differently, when gay men were touched by the male avatar, there were no differences between social and neutral areas (p=1.00) but touches on intimate area were considered more pleasurable than touches on neutral and social areas (p=.05, p=.012, respectively). In lesbian women, similarly to when men received touches by the female avatar, all the areas touched were considered equally (un)pleasant when touched by the male avatar (intimate vs neutral p=.99; intimate vs social p=.24, social vs neutral p=.84). When lesbian women were touched by the female avatar they considered equally (un)pleasant touches on all the areas.

| **Study 2 : Gay Men and Lesbian Women** | | | | | | | |
| --- | --- | --- | --- | --- | --- | --- | --- |
| **Touching_Avatar** | **Area** | **Gender** | **mean** | **SE** | **df** | **lower.CL** | **upper.CL** |
| Female | Intimate | Men | 47.9 | 3.45 | 54.9 | 41 | 54.8 |
| Male | Intimate | Men | 67.1 | 3.46 | 55.1 | 60.2 | 74.1 |
| Female | Neutral | Men | 49.5 | 2.68 | 79.5 | 44.2 | 54.8 |
| Male | Neutral | Men | 55.2 | 2.68 | 79.5 | 49.9 | 60.5 |
| Female | Social | Men | 50.6 | 2.93 | 71.5 | 44.8 | 56.5 |
| Male | Social | Men | 54.7 | 2.93 | 71.5 | 48.9 | 60.6 |
| Female | Intimate | Women | 57.7 | 3.45 | 54.9 | 50.8 | 64.6 |
| Male | Intimate | Women | 44.7 | 3.45 | 54.9 | 37.8 | 51.6 |
| Female | Neutral | Women | 53.3 | 2.68 | 79.5 | 48 | 58.7 |
| Male | Neutral | Women | 47.6 | 2.68 | 79.5 | 42.3 | 53 |
| Female | Social | Women | 61.3 | 2.93 | 71.5 | 55.4 | 67.1 |
| Male | Social | Women | 53.5 | 2.93 | 71.5 | 47.6 | 59.3 |

**Table S12.** Study 2 (Gay men and Lesbian women): means, SE, df, upper and lower CL for the interaction Gender:TouchingAvatar:Area found in the analysis of the (Un)pleasantness ratings.


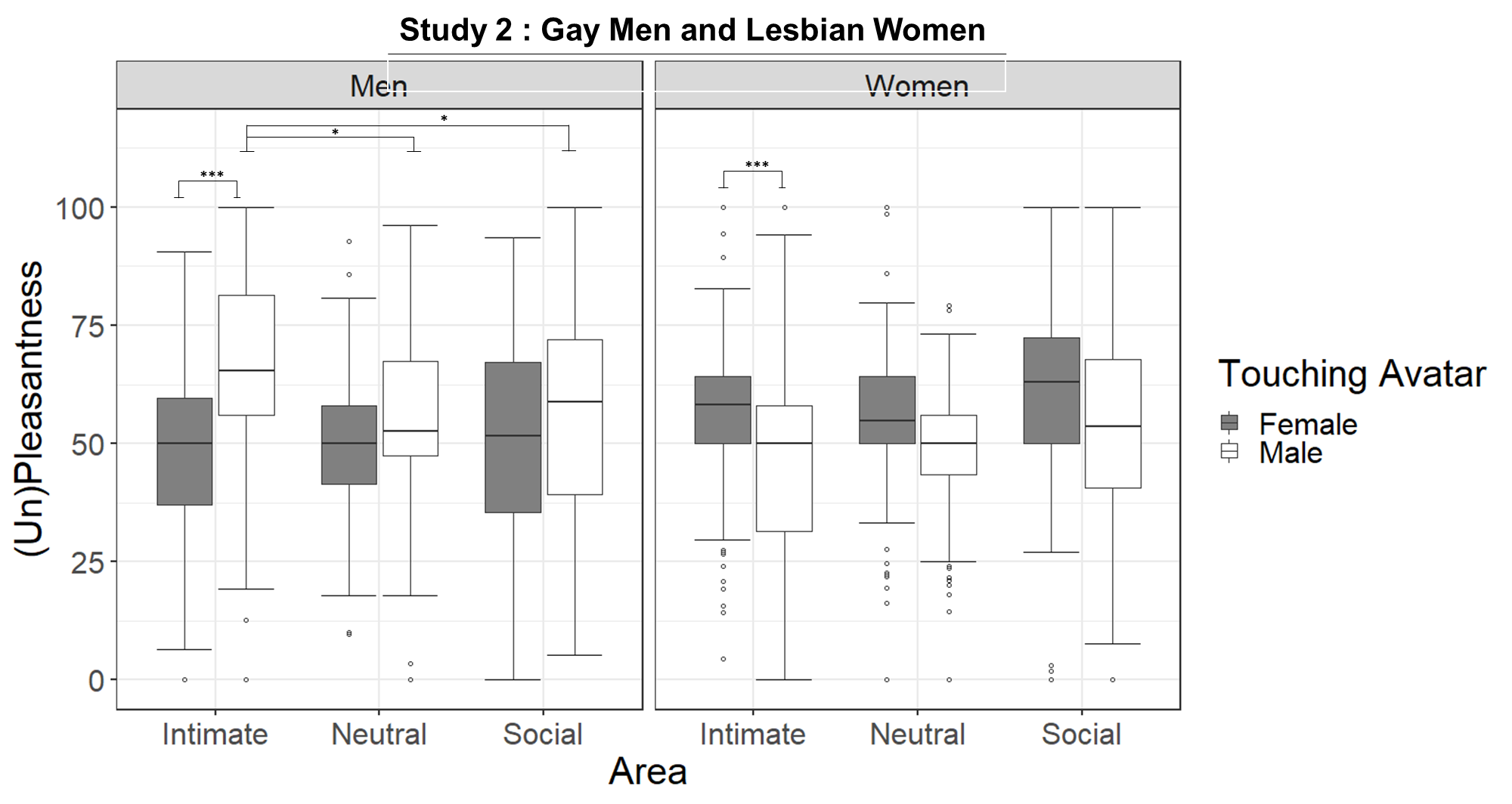


**Figure.S6.** Study 2 (Gay men and Lesbian women): Boxplots of the (Un)Pleasantness ratings. On each box, the central mark indicates the median, and the lower and upper hinges correspond to the 25th and 75th percentiles. The upper whisker extends to the largest value no further than 1.5 * IQR (inter-quartile range) from the hinge. The lower whisker extends to the smallest value at most 1.5 * IQR of the hinge. Points outside this range are shown individually. Signif.codes: '***' 0.001 '**' 0.01 '*' 0.05 '*’.

**Arousal**

The linear mixed effects model (R^2^_marginal_ = 0.064, R^2^_conditional_ =0.473) revealed a main effect of the Touching avatar and of the Area touched along with a 2-way interactions (see Table.S9, Table.S13 and Figure.S7). The main effect of the area (χ2(2)=23.125, p<.001) was accounted for by higher arousal for touches on intimate compared to neutral area (p<.001) and to social (p=.004) and equally arousing for neutral and social (p=.41). The interaction between Gender and the Touching avatar (χ2(2)=37.26, p<.001) showed that gay men considered as more arousing touches from male than from the female touching avatar (p<.001) and lesbian women more arousing touches from female than male touching avatar (p=.02).

| **Study 2 : Gay Men and Lesbian Women** | | | | | | |
| --- | --- | --- | --- | --- | --- | --- |
|  | **Area** | **Mean** | **SE** | **df** | **lower.CL** | **upper.CL** |
|  | Intimate | 64 | 2.42 | 44.1 | 59.1 | 68.9 |
|  | Neutral | 54 | 2.04 | 44.1 | 49.9 | 58.1 |
|  | Social | 56.8 | 2.46 | 44.1 | 51.8 | 61.8 |
| **Gender** | **Touching Avatar** | **Mean** | **SE** | **df** | **lower.CL** | **upper.CL** |
| Men | Female | 54.2 | 2.81 | 49.6 | 48.6 | 59.9 |
| Women | Female | 60.4 | 2.81 | 49.6 | 54.8 | 66.1 |
| Men | Male | 61.6 | 2.81 | 49.6 | 56 | 67.9 |
| Women | Male | 56.8 | 2.81 | 49.6 | 51.1 | 62.4 |

**Table S13.** Study 2 (Gay men and Lesbian women): means, SE, df, upper and lower CL for the main effect of Area and the interaction Gender:TouchingAvatar found in the analysis of the Arousal ratings


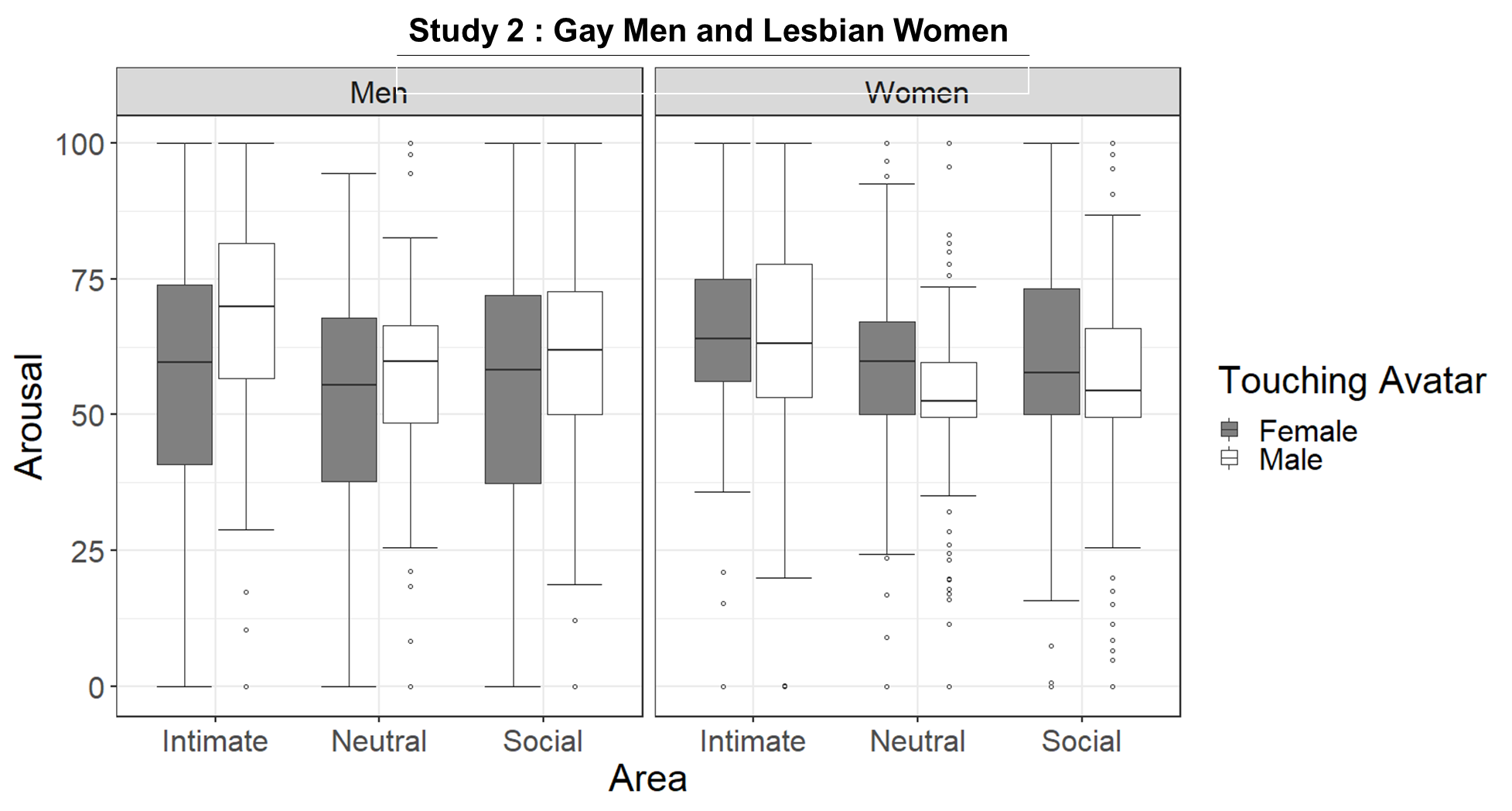


**Figure.S7.** Study 2 (Gay men and Lesbian women): Boxplot of the Arousal ratings for the interaction between Gender. Touching Avatar and Area. On each box, the central mark indicates the median, and the lower and upper hinges correspond to the 25th and 75th percentiles. The upper whisker extends to the largest value no further than 1.5 * IQR (inter-quartile range) from the hinge. The lower whisker extends to the smallest value at most 1.5 * IQR of the hinge. Points outside this range are shown individually.

**SCR**. In the table below, we show the estimated marginal means for the significant two-level interaction between Touching Avatar and Area.

| **Study 2 : Gay Men and Lesbian Women** | | | | | | |
| --- | --- | --- | --- | --- | --- | --- |
| **Touching_Avatar** | **Area** | **mean** | **SE** | **df** | **lower.CL** | **upper.CL** |
| Female | Intimate | 0.894 | 0.06 | 52.4 | 0.765 | 1.022 |
| Male | Intimate | 0.762 | 0.07 | 50.5 | 0.618 | 0.906 |
| Female | Neutral | 0.708 | 0.06 | 60.2 | 0.576 | 0.84 |
| Male | Neutral | 0.809 | 0.07 | 56.5 | 0.661 | 0.956 |
| Female | Social | 0.754 | 0.06 | 60.2 | 0.622 | 0.887 |
| Male | Social | 0.739 | 0.07 | 56.5 | 0.591 | 0.887 |

**Table S14.** Study 2(Gay men and Lesbian women): means, SE, df, upper and lower CL for the SCR at the factors combinations of Touching Avatar and Area.

**HR**. No significant difference was found. In the table below we show the estimated marginal means and confidence intervals for all the combinations between the factors’ levels.

| **Study 2 : Gay Men and Lesbian Women** | | | | | | | |
| --- | --- | --- | --- | --- | --- | --- | --- |
| **Touching_Avatar** | **Area** | **Gender** | **mean** | **SE** | **df** | **lower.CL** | **upper.CL** |
| Female | Intimate | Men | 8.08 | 0.262 | 45.0 | 7.55 | 8.61 |
| Male | Intimate | Men | 8.02 | 0.287 | 44.7 | 7.45 | 8.60 |
| Female | Neutral | Men | 7.96 | 0.264 | 46.7 | 7.43 | 8.50 |
| Male | Neutral | Men | 8.04 | 0.289 | 46.1 | 7.45 | 8.62 |
| Female | Social | Men | 8.15 | 0.264 | 46.7 | 7.62 | 8.69 |
| Male | Social | Men | 7.99 | 0.289 | 46.1 | 7.41 | 8.57 |
| Female | Intimate | Women | 7.89 | 0.269 | 45.0 | 7.35 | 8.43 |
| Male | Intimate | Women | 7.71 | 0.294 | 44.7 | 7.12 | 8.30 |
| Female | Neutral | Women | 7.76 | 0.271 | 46.7 | 7.22 | 8.31 |
| Male | Neutral | Women | 7.83 | 0.297 | 46.1 | 7.23 | 8.42 |
| Female | Social | Women | 7.89 | 0.271 | 46.7 | 7.34 | 8.43 |
| Male | Social | Women | 7.62 | 0.297 | 46.1 | 7.03 | 8.22 |

**Table S15**. Study 2 (Gay men and Lesbian women): HR (the number of heartbeats in a 6-second post-stimulus time window) means, SE, df, upper and lower CL for each combination between the factors’ levels.

**Correlations between physiological and behavioral measure**

Pearson’s correlation analysis was used to assess relations between SCR, HR, appropriateness, (un)pleasantness, arousal, and erogeneity. As for the other described analysis, only the seven body areas selected through the survey were used, grouped in the three areas (social, intimate neutral).To account for the repeated measurements on the same subjects, we estimated the Pearson’s coefficient between each variable using the subject averages ^10^P-values were corrected with the Holm-Bonferroni method^11^as implemented in the rcorr.adjust function from the R-package *RcmdrMisc* ^6^.

The physiological measures were not associated with the behavioral ratings in either study (Table.S16). In Study 1 (heterosexual men and women) a strong positive correlation (r = .734) was found between appropriateness and (un)pleasantness ratings (please note that on this scale, 0 represents the maximum unpleasantness and 100 the maximum pleasantness). This result was replicated in Study 2 (lesbian women and gay men) (*r* = .723) and is in line with the hypothesis that the hedonic properties of social touch are associated with how much a body region is considered as touchable by others. Indeed, the previous study by Suvilehto et al. ^12^showed that the ‘touchability index’ (i.e., the amount of body surface allowed to be touched), was linearly dependent on the emotional bond with the toucher and positively correlated with the experienced pleasantness of touch. A positive correlation between (un)pleasantness and erogeneity levels in Study 1 (*r* = .449) and Study 2 (*r* = .624) was also found, in keeping with what was found by Panagiotopoulou et al.^13^.

| **STUDY1**  Heterosexual Men and Women  N. of pairwise observations = 42 | (Un)pleasantness | Arousal | Erogeneity | SCR | HR |
| --- | --- | --- | --- | --- | --- |
| Appropriateness | **.734**** | .141 | .134 | .097 | .005 |
| (Un)pleasantness |  | .169 | **.449*** | .100 | .154 |
| Arousal |  |  | .161 | .116 | -.050 |
| Erogeneity |  |  |  | .151 | -.029 |
| SCR |  |  |  |  | -.030 |
| **STUDY2**  Gay Men and Lesbian Women  N. of pairwise observations = 42 | (Un)pleasantness | Arousal | Erogeneity | SCR | HR |
| Appropriateness | .723** | .015 | .370 | -.009 | .184 |
| (Un)pleasantness |  | .183 | .624** | -.040 | -.009 |
| Arousal |  |  | .254 | .049 | -.043 |
| Erogeneity |  |  |  | .123 | -.064 |
| SCR |  |  |  |  | .106 |

**Table S16**. Study 1 (heterosexual men and women) and Study 2 (lesbian women and gay men): Zero-order correlations for SCR, HR, appropriateness, (un)pleasantness, arousal, and erogeneity. Signif.codes: '***' 0.001 '**' 0.01 '*' 0.05 '*’.

|  |
| --- |

**Correlations between body ownership and vicarious touch**

Pearson’s correlation analysis was used to assess any relations between the Embodiment, the Vicarious Touch and the Control Items for Embodiment. As in the main analyses, item 1 and Item 2 were averaged to compute the Embodiment score; item 3 and 4 were averaged to compute the Control score; item 5 represented the Vicarious Touch score. P-values were corrected with the Holm’s procedure. A significant positive correlation between Embodiment and Vicarious Touch was found in Study 1 (r=0.458; Table.S17) and Study 2 (r=0.562; Table.S17); a negative association between Vicarious Touch and Control Questions for Embodiment was found in Study 2 (r=-.384) but not in Study 1 (r=.073, not significant).Taken together, the results suggest that the higher the feeling of owning the virtual body, the higher the feeling of being touched on it. Despite no inference can be made about the directionality of the relationship, this evidence is in line with previous suggestions of association between the self’s malleability and experience of vicarious sensations ^14^

| **STUDY1**  Heterosexual Men and Women  N. of pairwise observations = 42 | Control (Ownership) | Vicarious Touch |
| --- | --- | --- |
| Ownership | .047 | **.458*** |
| Control (Ownership) |  | .073 |
| **STUDY2**  Gay Men and Lesbian Women  N. of pairwise observations = 42 | Control (Ownership) | Vicarious Touch |
| Ownership | -.191 | **.562**** |
| Control (Ownership) |  | **-.384*** |

**Table S17**. Study 1 and Study 2: Zero-order correlations between Body Ownership, Body Ownership(Control) and Vicarious Touch.Signif.codes: '***' 0.001 '**' 0.01 '*' 0.05 '*’.

1.         Gregory Warnes, A. R., Bolker, B., Lumley, T., Johnson, R. C. & Gregory Warnes, M. R. *Package “gmodels” Title Various R Programming Tools for Model Fitting*. (2018).

2.         Field, A. & Website, C. *Discovering statistics using R*. https://uk.sagepub.com/en­gb/eur/discovering­statistics­using­r/book236067#contents (2012).

3.         Fusaro, M., Tieri, G. & Aglioti, S. M. Seeing pain and pleasure on self and others: Behavioural and psychophysiological reactivity in immersive virtual reality. *Journal of Neurophysiology* **116**, 2656–2662 (2016).

4.         Braithwaite, J. J., Derrick, D., Watson, G., Jones, R. & Rowe, M. *A Guide for Analysing Electrodermal Activity (EDA) & Skin Conductance Responses (SCRs) for Psychological Experiments*.

5.         Bates, D., Maechler, M., Bolker, B., Version, S. W.- & 2018, undefined. *Package “lme4.”* *researchgate.net* https://www.researchgate.net/profile/Sorour_Karimi/post/How_do_I_statistically_compare_cell_proliferation_rates2/attachment/5d119e3e3843b0b982582820/AS%3A773531587604482%401561435710646/download/lme4.pdf (2019).

6.         Fox, J. *et al.* Companion to Applied Regression: car.

7.         Lenth, R. *et al.* Emmeans: Estimated marginal means, aka least-squares means.

8.         Johnson, P. C. D. Extension of Nakagawa & Schielzeth’s R2GLMM to random slopes models. *Methods in Ecology and Evolution* **5**, 944–946 (2014).

9.         BARTON & K. MuMIn : multi-model inference. *http://r-forge.r-project.org/projects/mumin/* (2009).

10.        Irimata, K. Estimation of correlation coefficient in data with repeated measures.

11.        Stat, S. H.-J. & 1979, undefined. A simple sequentially rejective Bonferroni test procedure Scand.

12.        Suvilehto, J. T., Glerean, E., Dunbar, R. I. M., Hari, R. & Nummenmaa, L. Topography of social touching depends on emotional bonds between humans. *Proceedings of the National Academy of Sciences of the United States of America* **112**, 13811–13816 (2015).

13.        Panagiotopoulou, E., Filippetti, M. L., Gentsch, A. & Fotopoulou, A. Dissociable sources of erogeneity in social touch: Imagining and perceiving C-Tactile optimal touch in erogenous zones. *PLOS ONE* **13**, e0203039 (2018).

14.        Ward, J. & Banissy, M. J. Explaining mirror-touch synesthesia. *Cognitive Neuroscience* vol. 6 118–133 (2015).
